# A Novel Aquaporin Subfamily Imports Oxygen and Contributes to Pneumococcal Virulence by Controlling the Production and Release of Virulence Factors

**DOI:** 10.1128/mBio.01309-21

**Published:** 2021-08-17

**Authors:** Qingqing Hu, Huichun Tong, Jing Wang, Pupu Ge, Lin Zhu, Cuihua Liu, Jing-ren Zhang, Xiuzhu Dong

**Affiliations:** a State Key Laboratory of Microbial Resources, Institute of Microbiology, Chinese Academy of Sciences, Beijing, China; b University of Chinese Academy of Sciences, Beijing, China; c CAS Key Laboratory of Pathogenic Microbiology and Immunology, Institute of Microbiology, Center for Biosafety Mega-Science, Chinese Academy of Sciences, Beijing, China; d Savaid Medical School, University of Chinese Academy of Sciences, Beijing, China; e Center for Infectious Disease Research, School of Medicine, Tsinghua University, Beijing, China; Emory University School of Medicine

**Keywords:** *Streptococcus pneumoniae*, oxygen-transporting aquaporin, new subfamily of aquaporins, hydrogen peroxide production, ROS and RNS resistance, pneumolysin release, survival in macrophages, virulence

## Abstract

Aquaporins, integral membrane proteins widely distributed in organisms, facilitate the transport of water, glycerol, and other small uncharged solutes across cellular membranes and play important physiological roles in eukaryotes. However, characterizations and physiological functions of the prokaryotic aquaporins remain largely unknown. Here, we report that Streptococcus pneumoniae (pneumococcus) AqpC (Pn-AqpC), representing a new aquaporin subfamily possessing a distinct substrate-selective channel, functions as an oxygen porin by facilitating oxygen movement across the cell membrane and contributes significantly to pneumococcal virulence. The use of a phosphorescent oxygen probe showed that Pn-AqpC facilitates oxygen permeation into pneumococcal and Pn-AqpC-expressing yeast cells. Reconstituting Pn-AqpC into liposomes prepared with pneumococcal and Escherichia coli cellular membranes further verified that Pn-AqpC transports O_2_ but not water or glycerol. Alanine substitution showed that Pro232 in the substrate channel is key for Pn-AqpC in O_2_ transport. The deletion of Pn-*aqpC* significantly reduced H_2_O_2_ production and resistance to H_2_O_2_ and NO of pneumococci, whereas low-H_2_O_2_ treatment helped the ΔPn*-aqpC* mutant resist higher levels of H_2_O_2_ and even NO, indicating that Pn-AqpC-facilitated O_2_ permeation contributes to pneumococcal resistance to H_2_O_2_ and NO. Remarkably, the lack of Pn*-aqpC* alleviated cell autolysis, thus reducing pneumolysin (Ply) release and decreasing the hemolysis of pneumococci. Accordingly, the ΔPn-*aqpC* mutant markedly reduced survival in macrophages, decreased damage to macrophages, and significantly reduced lethality in mice. Therefore, the oxygen porin Pn-AqpC, through modulating H_2_O_2_ production and pneumolysin release, the two major pneumococcal virulence factors, controls the virulence of pneumococcus. Pn-AqpC orthologs are widely distributed in various pneumococcal serotypes, highlighting that the oxygen porin is important for pneumococcal pathogenicity.

## INTRODUCTION

Streptococcus pneumoniae (pneumococcus) is a major cause of community-acquired pneumonia, bacteremia, and meningitis (1, 2). It relies on multiple virulence factors, capsular polysaccharides (CPSs), pneumolysin (Ply), and H_2_O_2_, to transmit and escape the host’s innate immune system (1, 3–5). Distinguishingly, pneumococcus produces as well as resists high concentrations of H_2_O_2_ (6, 7). The ability to resist oxidative stress enables pneumococcus to survive against killing by host phagocytes (8, 9); particularly, intramacrophage survival is key to effective septic infection by pneumococcus (10). However, excess endogenous H_2_O_2_ also imposes oxidative stress on pneumococcus (11); therefore, effective efflux of H_2_O_2_ would be a way for detoxification. Recently, an aquaporin of Streptococcus oligofermentans, a close relative of pneumococcus, was reported to facilitate the efflux of cellular H_2_O_2_ (12). This provides a clue that pneumococcus may also employ aquaporins not only for the efflux of H_2_O_2_ for detoxification but also for virulence.

Aquaporins are integral membrane proteins found in diverse organisms (13–15). They facilitate the diffusion of water, glycerol, H_2_O_2_, ammonia, and other small uncharged solutes across cellular membranes and play important roles in physiological activities and diseases in eukaryotes (13, 15–19). Aquaporins are categorized into three major phylogenetic subfamilies: the classical water-transporting aquaporins (AQPs), the glycerol-transporting aquaglyceroporins (AQGPs), and the AQP supergene channel superaquaporins (SAQPs) (14). The three aquaporin subfamilies differ in their amino acid compositions of the aromatic/arginine constriction region (ar/R region), also known as the selective filter (20). However, only a few prokaryotic aquaporins have been studied regarding their physiological functions (21, 22).

Unexpectedly, we found that pneumococcus and some streptococcal pathogenic species encode a distinct aquaporin ortholog distantly related to the Escherichia coli glycerol facilitator GlpF, thus possibly representing a new aquaporin subfamily, which is tentatively assigned to the atypical aquaglyceroporins. Through the combination of physiological, biochemical, genetic, and pathogenicity assays, we demonstrated that pneumococcal AqpC (Pn-AqpC), a representative of the new aquaporin subfamily, facilitates O_2_ influx into pneumococcal and Pn-AqpC-expressing yeast cells and reconstituted proteoliposomes, thus functioning as an oxygen porin. By transporting O_2_, Pn-AqpC promoted pneumococcal H_2_O_2_ production, resistance to reactive oxygen species (ROS) and reactive nitrogen species (RNS), as well as the release of Ply, an important pneumococcal virulence factor (23). Importantly, Pn-AqpC elevated pneumococcal survival in macrophages and increased damage to macrophages, thus contributing significantly to pneumococcal pathogenicity in a murine pneumonia model. Pn*-aqpC* orthologs are widely distributed in all pneumococcal serotypes and capsule-free strains, implying that this oxygen porin could be a novel virulence-related protein.

## RESULTS

### Pneumococcus possesses an atypical aquaglyceroporin, Pn-AqpC, representing a novel aquaporin subfamily.

**FIG 1:**
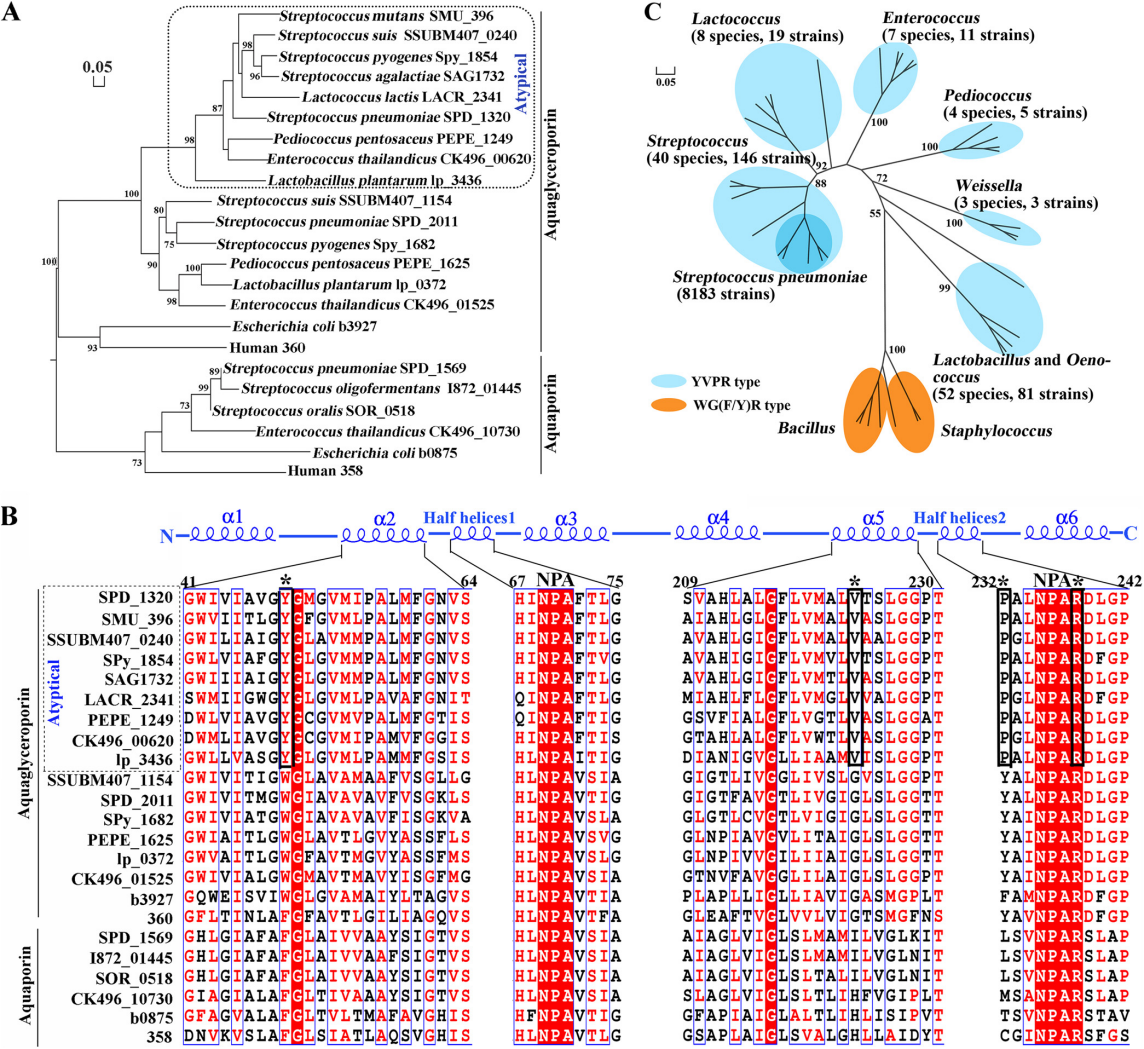
Phylogenetic analysis identifies a new subfamily of aquaporins with unique substrate-selective residues. (A) A phylogenetic tree based on the amino acid sequences of the aquaporin orthologs was constructed using the maximum likelihood method with 1,000 replicates. The bar of 0.05 represents evolutionary distance. A dotted line frames the new subfamily of aquaporins (atypical). (B) The amino acid sequences of the aquaporins in panel A were aligned using ClustalW. The E. coli GlpF (b3927) secondary structure (top panel) and the amino acid positions of D39 Pn-AqpC (SPD_1320) (top row of the bottom panel) are shown. Asterisks specify the ar/R region residues, and black lines frame YVPR of the atypical aquaglyceroporins. (C) Distribution of the new subfamily of aquaporins among *Lactobacillales*. Phylogenetic analysis was implemented as described above for panel A on at most five protein sequences of each genus. Numbers inside parentheses are those containing the YVPR-type aquaglyceroporins; branches within the dark blue pie represent pneumococcal strains.

Using the S. oligofermentans aquaporin So-AqpA (I872_01445) as a probe to query the genome of S. pneumoniae D39, an encapsulated serotype 2 strain, three genes (SPD_1320, SPD_1569, and SPD_2011) were hits at amino acid identities of 26%, 95%, and 31%, respectively. Phylogenetically, SPD_1569 clustered with So-AqpA and the water-facilitating aquaporin AqpZ of E. coli and thus was assigned as Pn-AqpA; SPD_2011 was clustered with the glycerol facilitator GlpF of E. coli and assigned as Pn-AqpB. However, SPD_1320 and some glycerol facilitators from other lactic acid bacteria clustered to form a separate branch distantly related to E. coli GlpF (Fig. 1A) and thus assigned as Pn-AqpC. Aquaporins in the Pn-AqpC-affiliated branch were tentatively named atypical aquaglyceroporins and could represent a new aquaporin subfamily. These aquaporins congruously possess YVPR as the substrate-selective residues (Fig. 1B; see also Fig. S1 in the supplemental material), which are distinct from F(H/I)XR in the water-transporting and WG(F/Y)R in glycerol-transporting aquaporins (Fig. 1B and C). Furthermore, the ar/R region diameter size in each Pn-AqpC monomer (Fig. S2A and B) was between those of the E. coli water (Fig. S2C)- and glycerol (Fig. S2D)-transporting aquaporins, implying a different substrate spectrum. Therefore, we investigated the physiological functions of Pn-AqpC, a representative of the new aquaporin subfamily.

10.1128/mBio.01309-21.2 FIG S1 Structural information on pneumococcal Pn-AqpC. (A) The characteristic Pn-AqpC ar/R region amino acid residues Tyr49, Val223, Pro232, and Arg238, situated at the substrate binding sites, are shown by sticks. (B) The membrane topology was analyzed using TMHMM software. The characteristic ar/R region amino acid residues are shown by blue letters, and the two NPA motifs are shown by orange letters. Download FIG S1, TIF file, 1.1 MB. Copyright © 2021 Hu et al. 2021 Hu et al. https://creativecommons.org/licenses/by/4.0/ This content is distributed under the terms of the Creative Commons Attribution 4.0 International license.

10.1128/mBio.01309-21.3 FIG S2 Structural homology modeling of pneumococcus Pn-AqpC and channel size comparison with E. coli AqpZ and GlpF. (A) The Pn-AqpC tetramer was constructed by structural homology modeling with SWISS-MODEL by automatically selecting aquaporin-10 (PDB accession no. 6F7H) as the template. (B to D) In each monomer, distances between Y49 and P232 (3.66 Å) and V223 and R238 (9.52 Å) in Pn-AqpC (B), F43 and T183 (5.30 Å) and H174 and R189 (4.20 Å) in E. coli AqpZ (PDB accession no. 1RC2) (C), and W48 and F200 (6.51 Å) and G191 and R206 (11.14 Å) in E. coli GlpF (PDB accession no. 1LDA) (D) were measured using PyMOL. Download FIG S2, TIF file, 2.4 MB. Copyright © 2021 Hu et al. 2021 Hu et al. https://creativecommons.org/licenses/by/4.0/ This content is distributed under the terms of the Creative Commons Attribution 4.0 International license.

### The absence of Pn-*aqpC* reduces H_2_O_2_ production but promotes the aerobic growth of pneumococcus.

To probe the physiological functions of Pn-AqpC, Pn-*aqpC* was deleted in S. pneumoniae D39 and its nonencapsulated mutant R6. By reference to *S. oligofermentans* So-AqpA that facilitates H_2_O_2_ permeation, the function of Pn-AqpC in H_2_O_2_ transport was first evaluated in R6 and its ΔPn*-aqpC* mutant carrying a specific cellular H_2_O_2_ reporter HyPer gene (24). However, the HyPer reporter detected similar H_2_O_2_ influx into ΔPn*-aqpC* and wild-type (WT) cells when provided exogenous H_2_O_2_ (Fig. S3A, bottom), thus excluding a role of Pn-AqpC in H_2_O_2_ permeation, whereas significantly lower fluorescence was found in ΔPn*-aqpC* cells when no exogenous H_2_O_2_ was provided (Fig. S3A, top), indicating reduced H_2_O_2_ production when Pn-AqpC is absent.

10.1128/mBio.01309-21.4 FIG S3 Pn-AqpC is not involved in H_2_O_2_ and H_2_O and glycerol transport. (A) The H_2_O_2_ reporter HyPer gene fused to the lactate dehydrogenase gene (*ldh*) promoter was inserted into pDL278 and then transformed into R6 and the ΔPn-*aqpC* mutant. The R6 WT-HyPer and ΔPn-*aqpC*-HyPer strains were statically grown in 10 ml BHI broth in a 100-ml flask. One milliliter of the mid-exponential-phase cells was washed twice and resuspended in 300 μl of PBS. One aliquot was challenged for 30 min with 0.5 mM H_2_O_2_ (+H_2_O_2_), leaving the other aliquot untreated (−H_2_O_2_). After 30 min of air exposure in the dark, the HyPer fluorescence of the cells was examined under a Leica TCS SP8 confocal laser scanning microscope system. Representative fluorescence (left) and corresponding differential interference contrast (DIC) (right) images from three independent experiments are shown. (B to E) Purified Pn-AqpC–10×His proteins were reconstituted into liposomes derived from the E. coli total lipid extract to generate proteoliposomes. To assay water permeability, 100 μl of Pn-AqpC-reconstituted proteoliposomes (B) and Pn-AqpC-devoid liposomes (C) were each rapidly mixed with 100 μl hyperosmolar sucrose to make a final osmotic gradient of 375 mosmol/liter, while to assay glycerol permeability, 100 μl of the glycerol solution was mixed with Pn-AqpC-inserted proteoliposomes (D) and Pn-AqpC-devoid liposomes (E) to make a final osmotic gradient of 375 mosmol/liter. Proteoliposome permeability was measured by monitoring the light scattering intensities using an SX20 stopped-flow spectrometer (Applied Photophysics, Surrey, UK) at 25°C under an emission wavelength of 600 nm. Increased light scattering indicates a vesicle volume decrease that is generated by higher external osmotic pressure-driven water efflux. While water will influx along with a transportable substrate and reswells the shrunken liposome leading to reduction of the light scattering intensity. The kinetics from 5 to 10 measurements were normalized and fitted to an exponential equation, from which the initial rates (*k*) of substrate permeation were calculated and are shown in the corresponding panels. Experiments were repeated three times, and averages ± standard errors (SE) from one independent assay are shown. Download FIG S3, TIF file, 1.8 MB. Copyright © 2021 Hu et al. 2021 Hu et al. https://creativecommons.org/licenses/by/4.0/ This content is distributed under the terms of the Creative Commons Attribution 4.0 International license.

**FIG 2:**
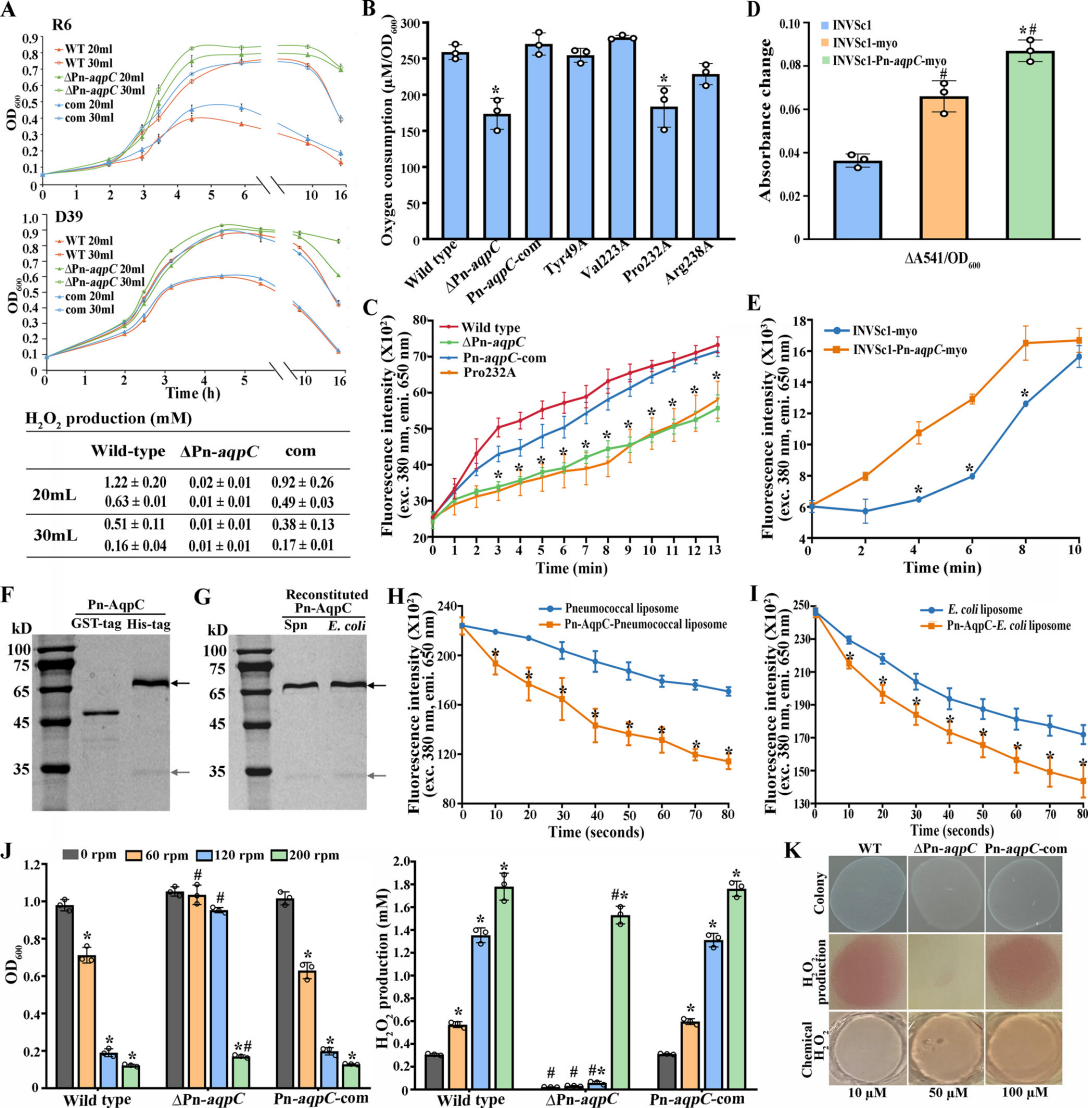
Pneumococcal Pn-AqpC acts as an oxygen porin. (A) Growth of R6 (top) and D39 (bottom) wild-type (WT), ΔPn-*aqpC*, and Pn-*aqpC*-complemented (com) strains cultured statically in 20 and 30 ml of BHI broth in a 100-ml flask. H_2_O_2_ (millimolar) accumulations in the stationary-phase cultures are listed in the table at the bottom, with those of strains R6 and D39 in the top and bottom rows, respectively. (B) An oxygen microsensor Oxy meter was used to measure the residual dissolved O_2_ in the stationary-phase cultures of the R6 wild type and its derivatives grown in 40 ml BHI broth in a 50-ml centrifuge tube. Oxygen consumption per OD_600_ of cell mass was calculated by comparison to 283 μM O_2_ in fresh medium. *, significantly different from other strains. (C) Mid-exponential-phase cultures of the R6 wild type and its derivatives were exposed to air, and the residual O_2_ in the culture was measured using a phosphorescent oxygen probe. *, significantly different from the wild-type and Pn-*aqpC*-complemented strains. (D) Protoplasts of S. cerevisiae INVSc1 and INVSc1 carrying the myoglobin gene (INVSc1-myo) or coexpressed with Pn-*aqpC* (INVSc1-Pn-*aqpC*-myo) were exposed to air, and the *A*_541_ increase per unit of biomass (Δ*A*_541_/OD_600_) was calculated. (E) The residual O_2_ contents in the culture were determined using a phosphorescent oxygen probe. # and *, significantly different from INVSc1 and INVSc1-myo, respectively. (F and G) The recombinant GST–Pn-AqpC–10×His protein was purified (GST-tag), digested with 100 U thrombin to remove the GST tag to obtain Pn-AqpC–10×His (His-tag) (F), and reconstituted into pneumococcal (Spn) and E. coli liposomes (G). The Pn-AqpC proteins were examined on a 12% SDS-PAGE gel. The protein ladder is shown at the left. Black and gray arrows indicate the macromolecular aggregate and monomer of Pn-AqpC protein, respectively. (H and I) Pn-AqpC facilitating O_2_ permeation across pneumococcal liposomes (H) and E. coli liposomes (I) was determined using a phosphorescent oxygen probe. *, the fluorescence intensity change at the respective time points was significantly different from that of the Pn-AqpC-devoid liposomes. (J) The R6 wild-type, ΔPn-*aqpC*, and Pn-*aqpC*-complemented (Pn-*aqpC*-com) strains were cultured with shaking at different speeds. The optical density at 600 nm (left) and H_2_O_2_ (millimolar) accumulating in stationary-phase cultures (right) were determined. # and *, significantly different from the wild-type and Pn-*aqpC*-com strains and the respective static cultures, respectively. (K, top and middle) Ten microliters of the mid-exponential-phase cultures in panel J was spotted onto a BHI agar plate and incubated in a 5% O_2_ environment for 10 h (top), and H_2_O_2_ was then determined (middle) as described in Materials and Methods. (Bottom) Chemical H_2_O_2_ with known concentrations was used as a reference. All experiments were conducted three times, and averages ± standard deviations (SD) (A, B, D, and J) or averages ± standard errors of the means (SEM) (C, E, H, and I) from one independent assay on triplicate samples are shown. For panels B to D and J, one-way ANOVA and Tukey’s test were performed; for panels E, H, and I, Student’s *t* test was performed (*P < *0.05).

Next, the H_2_O_2_ yields of the R6 and D39 wild-type strains and ΔPn*-aqp*C mutants were assayed in static cultures of 20 and 30 ml of brain heart infusion (BHI) broth in 100-ml flasks, respectively, which build gradient dissolved O_2_ levels. Surprisingly, the two ΔPn*-aqpC* mutants both achieved better growth and lower H_2_O_2_ yields than the wild-type strains in the two culture volumes (Fig. 2A), while the Pn*-aqpC*-complemented strains (Pn*-aqpC-*com) recovered the wild-type phenotype (Fig. 2A). This suggested that Pn-AqpC might facilitate the transmembrane diffusion of O_2_, a substrate for H_2_O_2_ formation. As similarly reduced cellular H_2_O_2_ and elevated aerobic growth were determined for the ΔPn-*aqpC* mutants of the nonencapsulated R6 and encapsulated D39 strains, the Pn-*aqpC* deletion-caused phenotype changes may not be related to the capsular polysaccharides; thus, strain R6 was investigated for the physiological functions *per se* of Pn-AqpC in the following experiments, except for animal studies.

### Pneumococcal Pn-AqpC facilitates O_2_ transport into cells.

To verify the function of Pn-AqpC in transporting O_2_, a dissolved oxygen microsensor Oxy meter (Unisense, Denmark) was used to measure the residual O_2_ contents in stationary-phase cultures of pneumococci growing in 40 ml BHI broth in a 50-ml centrifuge tube, and 1.5- to 1.6-fold-lower O_2_ consumption was determined for the R6 ΔPn*-aqpC* mutant than for the wild-type and Pn*-aqpC*-com strains (Fig. 2B). Similarly, using a phosphorescent oxygen probe, a 2.6-fold-lower O_2_ uptake rate within 3 min was determined for the ΔPn*-aqpC* mutant than for the wild-type strain, while the Pn*-aqpC*-com strain recovered O_2_ uptake levels of the wild type (Fig. 2C). Of note, the H_2_O_2_ yields in the wild type (122 ± 3 μM) and the ΔPn-*aqpC* mutant (18 ± 2 μM) were much lower than the theoretical stoichiometry (258 μM and 173 μM) calculated from the Oxy meter-measured oxygen consumption in the corresponding strains (258 ± 10 μM and 173 ± 21 μM). This indicates that other O_2_ consumption pathways are present, such as NADH oxidase (Nox) catalyzing the oxidization of NADH to NAD^+^ and H_2_O by using O_2_ as an electron acceptor (25). Therefore, we deleted *nox* in the wild type and the ΔPn-*aqpC* mutant, which reduced O_2_ consumption by 30 ± 3.5 and 34 ± 5.8 μM, respectively. Nevertheless, there should have been other unknown pathways consuming the remaining 106 μM and 121 μM O_2_ in the wild type and the ΔPn-*aqpC* mutant, respectively. Moreover, Pn-*aqpC* deletion did not alter the expression of *spxB* and *lctO*, which encode the two major H_2_O_2_ production enzymes pyruvate oxidase and lactate oxidase, respectively (Fig. S4). This indicates that the reduced H_2_O_2_ production in the ΔPn-*aqpC* mutant was due to decreased O_2_ influx instead of reduced expression of the H_2_O_2_ production genes.

10.1128/mBio.01309-21.5 FIG S4 Effects of Pn-*aqpC* deletion on the expression of *lytA*, *psaA*, *pspC*, *spxB*, and *lctO* in the pneumococcus R6 strain. Total RNAs were extracted from statically grown mid-exponential-phase R6 cells using TRIzol reagent. After quality confirmation on a 1% agarose gel, cDNAs were generated from 2 μg of total RNA with random primers using Moloney murine leukemia virus reverse transcriptase. Quantitative reverse transcription-PCR (RT-qPCR) was implemented using the corresponding primers listed in Table S2 in the supplemental material. Experiments were repeated 3 times on triplicate samples. The transcript copies were calculated per 1,000 16S rRNA copies, and the averages ± SD from three independent experiments are shown. Download FIG S4, TIF file, 0.1 MB. Copyright © 2021 Hu et al. 2021 Hu et al. https://creativecommons.org/licenses/by/4.0/ This content is distributed under the terms of the Creative Commons Attribution 4.0 International license.

The O_2_-facilitating function of Pn-AqpC was further verified by the coexpression of Pn-*aqpC* and the sperm whale myoglobin (Mb) gene in Saccharomyces cerevisiae INVSc1. Single-Mb-gene-expressing INVSc1-myo and INVSc1 strains were used as controls. In addition, an INVSc1-Pn*-aqpC*-*gfp* strain carrying a super folder green fluorescent protein (GFP) gene (sf*gfp*) fusion to Pn*-aqpC* was constructed. The expressions of Pn-AqpC and Mb in S. cerevisiae were verified by Western blotting (Fig. S5A), and the cytoplasmic membrane localization of heterologously expressed Pn-AqpC in S. cerevisiae was confirmed via confocal microscopy examination (Fig. S5B). By measuring the characteristic oxymyoglobin (MbO_2_) absorption at 541 nm in yeast protoplasts (26) (Fig. S5C) and purified MbO_2_ (Fig. S5D), a significant MbO_2_ increase (Δ*A*_541_/optical density at 600 nm [OD_600_]) was found in Pn*-aqpC*-expressing yeast compared to Pn-*aqpC*-devoid INVSc1 (Fig. 2D). Accordingly, the phosphorescent oxygen probe detected a more rapid decrease of the cultural O_2_ content of INVSc1-Pn*-aqpC*-myo than that of INVSc1-myo (Fig. 2E). These results collectively showed that Pn-AqpC facilitates oxygen permeation.

10.1128/mBio.01309-21.6 FIG S5 Verification of Pn-*aqpC* and myoglobin expression in yeast (A and B) and absorption spectra of Pn-*aqpC*-overexpressing yeast (C) and purified hemoglobin (D). (A) An N-terminally Flag-fused Pn*-aqpC* gene and a C-terminally 6×His-fused myoglobin gene (myo) from sperm whale (Physeter macrocephalus) were coexpressed in Saccharomyces cerevisiae INVSc1 to obtain strain INVSc1-Pn*-aqpC-*myo, and the myoglobin gene singly expressed strain INVSc1-myo was used as a background control. After 16 h of culture in SD medium lacking Ura and Leu (SD-Ura-Leu) supplemented with 2% galactose, the two strains were lysed. The same amounts of the cell lysates were used to detect the Pn-AqpC and myoglobin expression by Western blotting using anti-Flag and anti-His antibodies, respectively. (B) Confocal examination shows the cellular localization of Pn-AqpC in S. cerevisiae INVSc1-Pn-*aqpC*-*gfp* that carried the green fluorescent protein gene fusion to Pn-AqpC. Representative differential interference contrast (left) and GFP fluorescence (right) images are shown. (C) The same amounts of mid-exponential-phase INVSc1-Pn*-aqpC-*myo (left) and INVSc1-myo (middle) cells grown in SD-Ura-Leu galactose medium and INVSc1 (right) cells grown in yeast extract-peptone-dextrose (YPD) medium were prepared as protoplasts. The protoplasts were resuspended in isotonic buffer (1.2 M sorbitol, 50 mM magnesium acetate, 10 mM CaCl_2_), deoxygenated by 7 cycles of vacuum and nitrogen gas flushing, and then fully air exposed with an oxygen pump. The air-exposed cells were dispersed into a 96-well plate, and the absorption spectra were monitored at wavelengths from 300 to 650 nm for 7 min at 60-s intervals using a Synergy H4 hybrid multimode microplate reader (BioTek, Winooski, VT). Experiments were repeated three times, and representative spectra are shown. (D) Hemoglobin (Macklin, Shanghai, China) was dissolved in PBS containing 5% sodium ascorbate to a final concentration of 1 mg/ml, and oxygen was removed by 7 cycles of vacuum-nitrogen flush. The deoxygenated hemoglobin was then air exposed for 15 min using an oxygen pump to obtain oxygenated hemoglobin. The absorption spectra of deoxygenated and oxygenated hemoglobin were monitored under wavelengths of 300 to 700 nm using the Synergy H4 hybrid multimode microplate reader (BioTek). The red dotted line frames the characteristic absorption of oxygenated hemoglobin at 541 nm. Experiments were repeated three times, and a representative spectrum is shown. Download FIG S5, TIF file, 1.4 MB. Copyright © 2021 Hu et al. 2021 Hu et al. https://creativecommons.org/licenses/by/4.0/ This content is distributed under the terms of the Creative Commons Attribution 4.0 International license.

### Pn-AqpC facilitates oxygen transport across proteoliposomes.

To further confirm that Pn-AqpC facilitates the transport of O_2_ and other substrates, the recombinant glutathione *S*-transferase (GST)–Pn-AqpC–10×His protein was purified in the detergent octylglucoside (OG), and the GST tag was then removed (Fig. 2F). Two Pn-AqpC–10×His protein bands of ∼32 kDa and ∼68 kDa were identified as Pn-AqpC by liquid chromatography-tandem mass spectrometry (LC-MS/MS) analysis (Fig. 2F and G; Table S1). Compared to the E. coli AqpZ tetramers that hardly dissociated with 1% SDS due to the strong hydrophobic characteristics (27), the 68-kDa protein was assumed to be a macromolecular aggregate, while the 32-kDa protein was assumed to be a monomer. The purified Pn-AqpC–10×His protein was reconstituted into the membrane lipid of pneumococci and E. coli (Fig. 2G). The phosphorescent oxygen probe was then wrapped within the proteoliposomes and Pn-AqpC-devoid liposomes to detect oxygen contents. This detected 3.2- and 1.5-fold-higher O_2_ influx in the first 40 s into the pneumococcal and E. coli proteoliposomes than the respective Pn-AqpC-devoid liposomes, respectively (Fig. 2H and I). Notably, 3-fold-higher O_2_ influx was determined for Pn-AqpC-devoid E. coli than for the pneumococcal liposomes within 80 s (Fig. 2H and I). These results demonstrated that Pn-AqpC sped up O_2_ permeation across the cellular membrane, and the pneumococcal membrane appears to have lower O_2_ permeability than that of E. coli.

10.1128/mBio.01309-21.8 TABLE S1 LC-MS/MS identification of Pn-AqpC–10×His proteins. Download Table S1, DOCX file, 0.02 MB. Copyright © 2021 Hu et al. 2021 Hu et al. https://creativecommons.org/licenses/by/4.0/ This content is distributed under the terms of the Creative Commons Attribution 4.0 International license.

Using a stopped-flow apparatus, Pn-AqpC facilitating water and glycerol permeation was measured based on osmosis-driven permeability. However, similar initial rates (*k*) were determined for proteoliposomes and Pn-AqpC-devoid liposomes in water and glycerol permeation (Fig. S3B to E). Therefore, Pn-AqpC specifically facilitates the permeation of O_2_ but not water or glycerol.

### Pn-AqpC acts as a prominent oxygen facilitator under lower oxygen levels.

Given that O_2_, particularly at higher concentrations, permeates freely through the cytoplasmic membrane, the range of O_2_ levels wherein Pn-AqpC plays a role in facilitating O_2_ was tested by growing the R6 wild-type, ΔPn*-aqpC* mutant, and Pn-*aqpC*-com strains under gradient shaking speeds. Although the three strains exhibited similar growth rates under static and 200-rpm shaking conditions, the ΔPn*-aqpC* mutant grew slightly and markedly better under 60- and 120-rpm shaking conditions (Fig. 2J, left). Accordingly, significantly lower levels of H_2_O_2_ were produced in the ΔPn*-aqpC* mutant cultured under static, 60-rpm, and 120-rpm conditions, but similar H_2_O_2_ levels were generated in 200-rpm shaking cultures of wild-type, ΔPn*-aqpC* mutant, and Pn-*aqpC*-com strains (Fig. 2J, right). This shows that Pn-AqpC exerts an O_2_ facilitator role when the bacterium lives under lower O_2_ levels. Next, the role of Pn-AqpC in the H_2_O_2_ production of pneumococci under 5% O_2_ was tested by mimicking the O_2_ concentrations in the human lower respiratory tract within the mucus layer or in close contact with pulmonary epithelial cells (11). The same amounts of the wild-type, ΔPn*-aqpC* mutant, and Pn-*aqpC*-com cultures were spotted onto a BHI agar plate and incubated in an O_2_ control *in vitro* glove box (Coy Laboratory Products). Compared with the wild-type and complemented strains, the ΔPn*-aqpC* mutant produced almost undetectable H_2_O_2_ under 5% O_2_ (Fig. 2K); therefore, Pn-AqpC could play an important role in pneumococcal infection by facilitating O_2_ import for H_2_O_2_ production, one of the pneumococcal virulence factors.

### The substrate-selective residue Pro232 is essential for Pn-AqpC in facilitating O_2_ permeation.

To determine the key substrate-selective residues for Pn-AqpC in O_2_ transport, alanine substitution was performed for each YVPR on the shuttle vector pDL278-Pn*-aqpC* and then transformed into the ΔPn*-aqpC* mutant to obtain the Tyr49A, Val223A, Pro232A, and Arg238A strains. The four mutants and the Pn*-aqpC*-com strain were grown in 10 ml BHI broth, and H_2_O_2_ yields in the stationary-phase cultures were used as a proxy for O_2_ uptake. Threefold-reduced H_2_O_2_ yields were determined for the Pro232A mutant (0.48 ± 0.39 mM) compared with the Pn*-aqpC*-com strain (1.46 ± 0.21 mM), whereas no significant change was observed for the Tyr49A (1.38 ± 0.17 mM), Val223A (1.41 ± 0.18 mM), and Arg238A (1.27 ± 0.26 mM) strains.

Furthermore, using the oxygen microsensor Oxy meter, 1.4-fold-lower O_2_ consumption for the Pro232A mutant was determined than for the wild-type and Pn*-aqpC*-com strains (Fig. 2B). The phosphorescent oxygen probe also measured 3.3- and 2.3-fold-lower O_2_ consumption rates within 3 min for the Pro232A mutant than for the wild-type and Pn*-aqpC*-com strains, respectively (Fig. 2C). These results revealed that Pro232 is the key residue of Pn-AqpC in O_2_ transport.

### Elevated Pn-AqpC protein contents occur in aerobic cultures.

**FIG 3:**
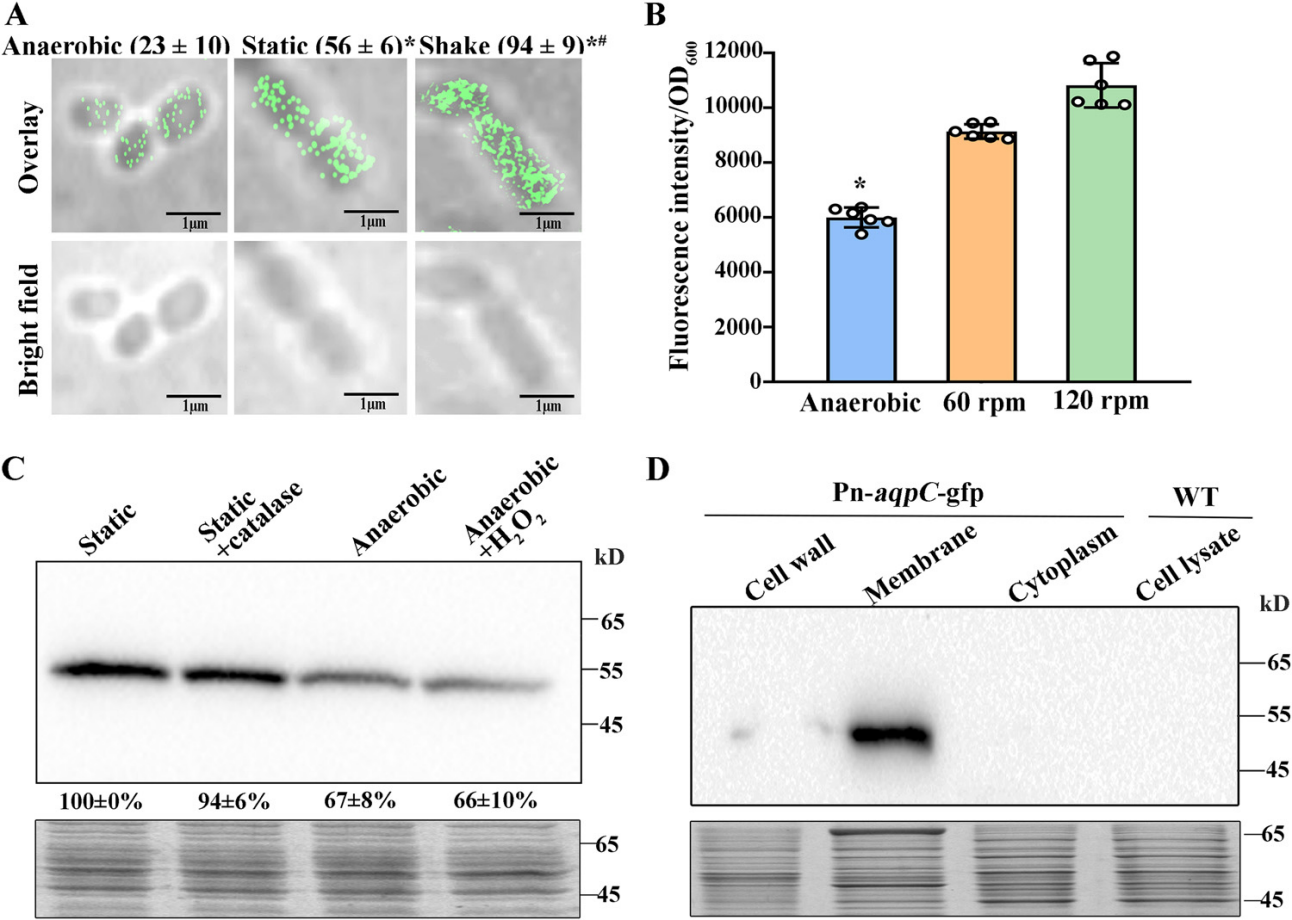
Oxygen induces Pn-*aqpC* expression. (A) PALM imaging to assay Pn-AqpC protein expression in the R6 Pn-AqpC-mMaple3 strain grown anaerobically, statically, or with shaking at 120 rpm with the addition of catalase. The mMaple3 fluorescence of mid-exponential-phase cells was observed after 30 min of air exposure in the dark. Pn-AqpC protein numbers were quantified in 18 representative cells, and averages ± SD per cell are shown in parentheses. * and #, significantly different from anaerobically and statically grown cells, respectively (*P < *0.05 by one-way ANOVA with Tukey’s test). (B) GFP fluorescence of the mid-exponential-phase cultures was measured in R6 Pn*-aqpC*-*gfp* cells cultured anaerobically and shaken with the addition of catalase after air exposure in the dark. The experiments were repeated three times, and averages ± SD from one independent experiment with sextuplicate samples are shown. *, significantly different from the shaking culture cells (*P < *0.05 by one-way ANOVA with Tukey’s test). (C, top) Western blot assay of the expression of GFP-tagged Pn-AqpC in the R6 Pn*-aqpC*-*gfp* strain grown statically with or without 1 kilounit (KU)/ml catalase and anaerobically with or without 40 μM H_2_O_2_ treatment. Band intensities were measured using ImageJ and are shown as percentages of that in static culture. Averages ± SD from three experiments are shown. (Bottom) Total protein separated on an SDS-PAGE gel and used as the protein loading control. (D, top) Statically grown Pn*-aqpC*-*gfp* cells were fractionated into cell wall, membrane, and cytoplasmic fractions and subjected to Western blotting using anti-GFP monoclonal antibody. The cell lysate of the wild-type strain (WT) was used as a negative control. (Bottom) SDS-PAGE gel of total protein in each fraction used as the protein loading control. Representative results from three independent experiments are shown in panels A, C, and D.

Given the role of Pn-AqpC in O_2_ transport, its synthesis in response to O_2_ was determined. Using the photoactivated localization microscopy (PALM) superresolution imaging technique (28), numbers of Pn-AqpC proteins per cell were quantified in anaerobically, statically, and 120-rpm-shaking-grown cultures of the Pn*-aqpC*-mMaple3 strain, which carried an mMaple3 protein (29) fusion at the C terminus of Pn-*aqpC*. Figure 3A shows representative PALM images with mMaple3 fluorescence signals; each image included a 2- to 3-cell-constituted cell chain, the typical morphology of pneumococcus. PALM data analysis indicated that the average numbers of Pn-AqpC protein molecules per cell were 23 ± 10 in anaerobic, 56 ± 6 in static, and 94 ± 9 in shaking cultures (Fig. 3A). Catalase treatment did not reduce the Pn-AqpC protein numbers (41 ± 21) in static culture; thus, O_2_, but not H_2_O_2_, seems to induce Pn-AqpC expression.

Oxygen-induced Pn-AqpC expression was further verified by the GFP reporter strain Pn*-aqpC*-*gfp*. GFP fluorescence intensities showed a pattern of in anaerobic culture <60 rpm shaking culture <120 rpm shaking culture (Fig. 3B), whereas neither catalase treatment of the static culture nor H_2_O_2_ pulsing of the anaerobic culture changed the O_2_-level-related Pn-AqpC abundances (Fig. 3C). Pn-AqpC was detected exclusively in the cellular membrane fraction (Fig. 3D), confirming its membrane protein identity as predicted by TMHMM (Fig. S1B). These findings indicated that O_2_ induces the synthesis of Pn-AqpC.

### The absence of Pn-AqpC reduces pneumococcal resistance to H_2_O_2_ and NO as well as Ply release.

**FIG 4:**
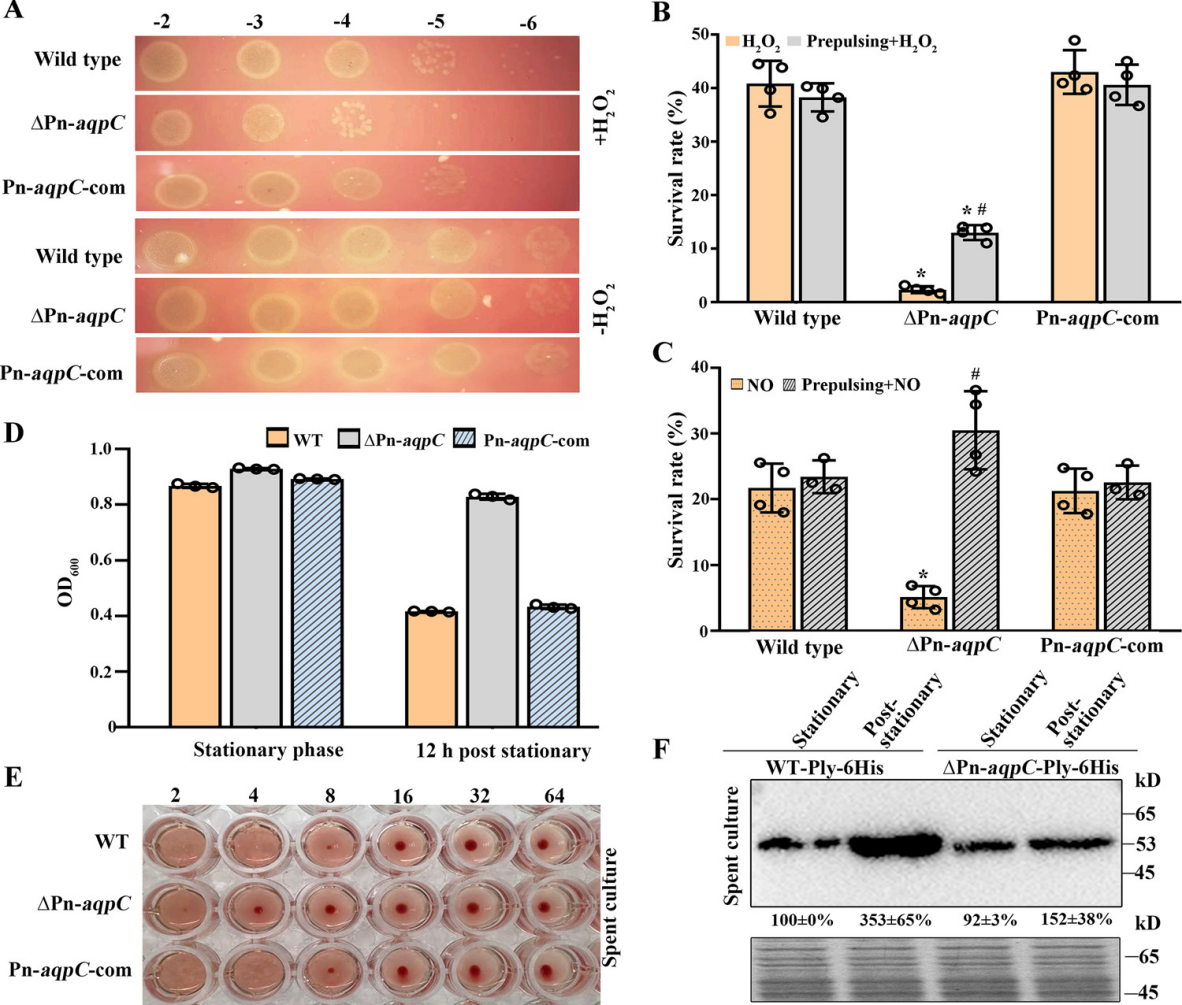
Absence of Pn-AqpC reduces oxidative resistance and Ply release of pneumococcus. (A) The same amounts of statically grown R6 and its derivatives were 10-fold serially diluted and spotted onto BHI agar containing 10 mM H_2_O_2_ or not. Representative growth results from triplicates are shown. (B and C) Strains in panel A were either directly treated or prepulsed with 40 μM H_2_O_2_ before being treated with 10 mM H_2_O_2_ (B) or 5 mM NO (C). Survival rates are calculated based on CFU. * and #, significantly different from the wild-type and complemented strains and the H_2_O_2_ or NO directly treated ΔPn*-aqpC* mutant, respectively (*P < *0.05 by one-way ANOVA with Tukey’s test). (D) Cell autolysis in the stationary and 12-h-post-stationary phases of the strains in panel A was assayed by measurement of the culture OD_600_. (E) Erythrocytes were incubated with the diluted (fold indicated at the top) 12-h-post-stationary-phase spent cultures of the strains in panel D. Hemolysis was examined after 30 min of incubation at 37°C. (F) Western blot assay of Ply amounts released from the R6 wild type (WT) and the ΔPn*-aqpC* mutant. 6×His-tagged Ply was expressed in the two strains, which were cultured statically until the stationary and 12-h-post-stationary phases. (Top) The same amounts of 10-kDa ultrafiltration-concentrated cultures were separated on an SDS-PAGE gel, and anti-His antibody was used to detect 6×His-tagged Ply. Band intensities were quantified using ImageJ software, and Ply contents are shown as percentages of the WT Ply-6×His strain (100%) in the stationary-phase spent culture. (Bottom) Total proteins separated on an SDS-PAGE gel were included as a protein loading control. All experiments were executed three times, and averages ± SD from one independent assay on triplicate or quadruplicate samples are shown. Representative results from three independent experiments are shown in panels A, E, and F.

Given that endogenous H_2_O_2_ assists pneumococcus in resisting oxidative stress (7), the ΔPn-*aqpC* mutant reducing H_2_O_2_ resistance was presumed to be due to lower H_2_O_2_ production. As expected, a lower MIC of H_2_O_2_ was determined for the ΔPn-*aqpC* mutant (5 mM) than for the wild-type strain (8 mM). Consistently, the growth of ΔPn-*aqpC* in a BHI plate containing 10 mM H_2_O_2_ occurred only at a 10^−5^ dilution compared with the 10^−6^ dilutions of the wild-type and Pn-*aqpC*-com strains (Fig. 4A). Moreover, only 2.3% of ΔPn-*aqpC* mutant cells survived the challenge with 10 mM H_2_O_2_, compared with survival rates of >40% for the wild-type and Pn-*aqpC*-com strains (Fig. 4B). However, 40 μM H_2_O_2_ prepulsing significantly increased 10 mM H_2_O_2_ survival of the ΔPn-*aqpC* mutant (Fig. 4B), indicating that Pn-AqpC-promoted endogenous H_2_O_2_ production makes pneumococcus withstand exogenous H_2_O_2_ challenge.

Next, the role of Pn-AqpC in pneumococcal resistance to NO, another oxidant, was assayed as macrophages employ NO-dependent bactericidal mechanisms to clear infecting bacteria (8). Only 5% of the ΔPn-*aqpC* cells survived 5 mM NO, compared to about 20% survival of the wild-type and Pn-*aqpC*-com strains (Fig. 4C), suggesting the involvement of Pn-AqpC in NO resistance. Given that the proteins involved in H_2_O_2_ resistance also assist E. coli in resisting NO (30), we used 40 μM H_2_O_2_ to prepulse the three strains. H_2_O_2_ prepulsing increased the NO survival of the ΔPn-*aqpC* mutant by 6-fold but had no effect on the survival of the wild-type and Pn-*aqpC*-com strains (Fig. 4C). This shows that lower H_2_O_2_ levels induce cross-protection of pneumococci from NO stress.

Stationary-phase cells of pneumococci are usually autolyzed and thus release Ply (31), a major virulence factor. The deletion of Pn-*aqpC* appeared to significantly alleviate cell autolysis (Fig. 4D) and so may also reduce Ply release and the hemolytic activity of pneumococcus. To test this, 12-h-post-stationary-phase spent cultures of the wild-type, Pn-*aqpC* deletion, and complemented strains were 2-fold serially diluted, and horse red blood cells were added. Complete erythrocyte lysis was observed in ≤4-fold dilutions of the wild-type and Pn-*aqpC*-com cultures, but only partial hemolysis occurred in the 2-fold-diluted ΔPn-*aqpC* culture (Fig. 4E). Consistently, about 2.3-fold less Ply protein was detected in the 12-h-post-stationary-phase spent culture of the ΔPn-*aqpC* mutant (Fig. 4F). This shows that Pn-*aqpC* deletion reduces cell lysis as well as Ply release.

### Deletion of Pn*-aqpC* reduces pneumococcal survival in macrophages and damage to macrophages.

**FIG 5:**
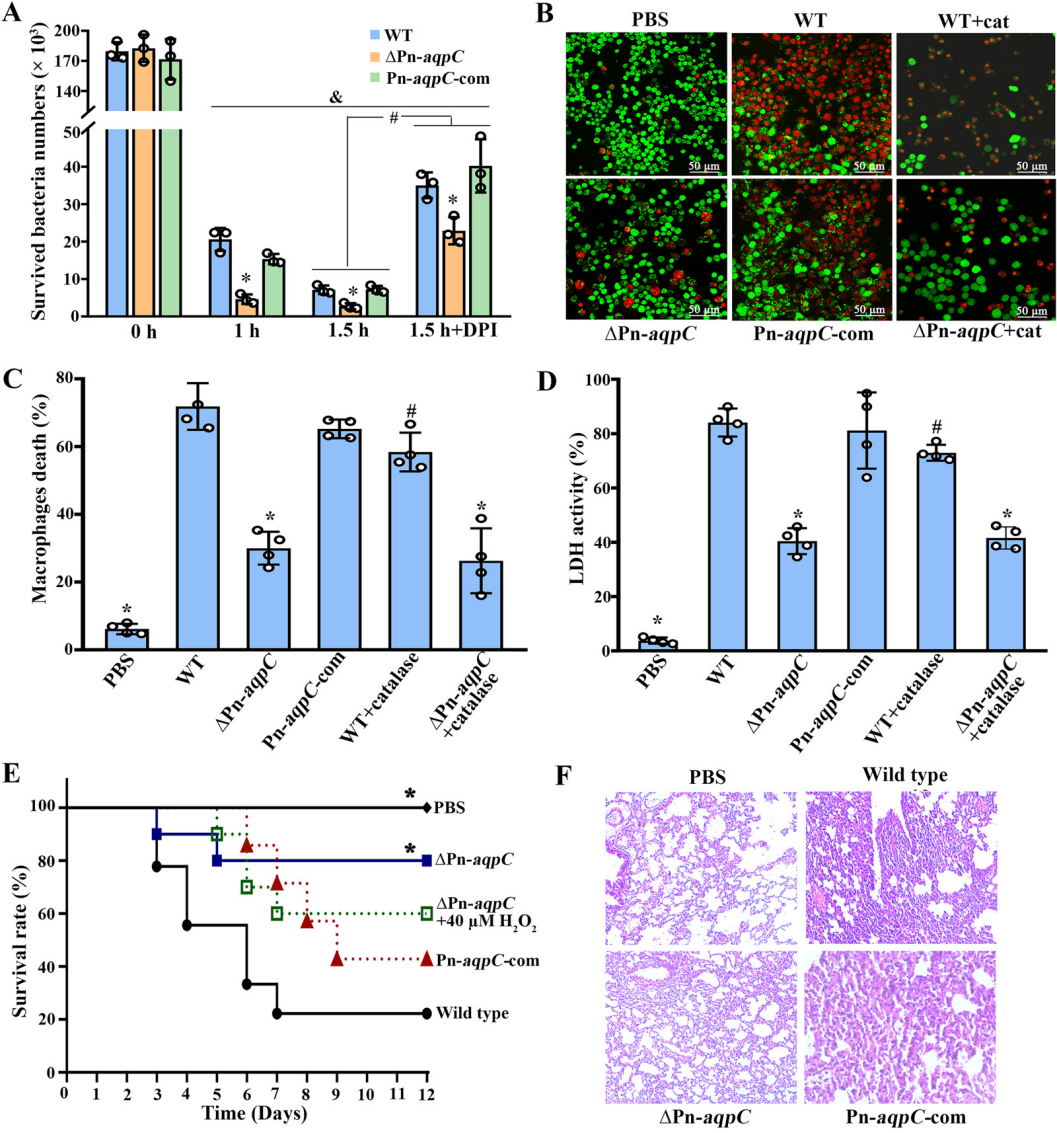
Deletion of Pn-*aqpC* reduces pneumococcal survival and damage to macrophages and significantly attenuates virulence to mice. (A) Pneumococcal survival in macrophages was assayed by coincubation of 1 × 10^5^ RAW 264.7 cells with the R6 wild type (WT) and derivatives at an MOI of 100:1. After 1 h of incubation, bacterial cells in the culture were removed, those attached to and internalized in macrophages were recorded, and this time point was set as 0 h. After additional 1- and 1.5-h (total, 2- and 2.5-h) incubations in fresh medium containing antibiotics, the pneumococcal cells that survived within macrophages were counted. The nitric oxide (NO) synthetase inhibitor DPI was added to reduce NO production by macrophages. (B to D) Using the same approach as the one described above for panel A, the contribution of Pn-AqpC to R6’s damage to macrophages was evaluated. (B) Live (green)/dead (red) cell staining of macrophages. The addition of 1 KU/ml catalase (cat) was used to assay the role of H_2_O_2_. (C) After 16 h of antibiotic treatment, macrophage death (percent) was calculated. (D) Bacterial damage to macrophages was also evaluated by leaked lactate dehydrogenase (LDH) activities. &, significantly different from the respective strains at 0 h (A); *, significantly different from the wild-type and Pn*-aqpC*-com strains; #, significantly different from that without DPI treatment (A) or the respective strain without catalase addition (C and D) (*P *<* *0.05 by one-way ANOVA and Tukey’s test). (E) BALB/c mice (*n* = 10; female) were intratracheally infected with 1 × 10^7^ CFU of statically grown D39 wild-type and ΔPn*-aqpC* strains with or without 40 μM H_2_O_2_ pretreatment and the Pn*-aqpC*-com strain. PBS-administered mice were included as controls. Survival of the infected mice was monitored for up to 12 days, and representative survival curves from two independent experiments are shown. *, significantly different from the wild-type and Pn*-aqpC*-com strains (*P < *0.05 by a log rank Mantel-Cox test). (F) Histopathological observation of the lung tissues of mice that survived PBS administration and ΔPn*-aqpC* infection and those that died from wild-type and Pn*-aqpC*-com infections.

Macrophages, the first line of defense of the human immune system, utilize reactive oxygen and nitrogen species to kill invading microbes (8, 9). Given the reduced H_2_O_2_ production of the ΔPn-*aqpC* mutant, the effect of the Pn*-aqpC* deletion on pneumococcal survival in macrophages was investigated. First, 1 × 10^5^ macrophage RAW 264.7 cells were exposed to the nonencapsulated R6 wild-type, Pn*-aqpC* deletion, and complemented strains at a multiplicity of infection (MOI) of 100:1. After 1 h of incubation, the bacterial cells attached to and internalized into macrophages were counted, and after additional 1-h and 1.5-h incubations, surviving pneumococcal cells within macrophages were counted. Although the numbers of viable bacterial cells of the three strains all significantly decreased, 4.5- and 3-fold-lower survival rates of the ΔPn*-aqpC* mutant were determined after additional 1-h and 1.5-h incubations, respectively, than for the wild-type and Pn*-aqpC*-com strains (Fig. 5A). This validates the contribution of Pn-AqpC to pneumococcal survival in macrophages.

To query whether the reduced macrophage survival is caused by the increased NO sensitivity of the ΔPn-*aqpC* mutant, diphenyleneiodonium chloride (DPI), an inhibitor of inducible nitric oxide synthase (iNOS) (32), was used to inhibit NO production by macrophages. The addition of DPI increased the number of living cells 4.9- and 5.6-fold for the wild-type and Pn-*aqpC*-com strains, respectively, whereas it enhanced the survival of the ΔPn-*aqpC* mutant 8.4-fold in macrophages (Fig. 5A), indicating that Pn-AqpC-conferred pneumococcus oxidative stress resistance assists its survival in macrophages.

Given that H_2_O_2_ induces macrophage death (33), the role of the deletion of Pn*-aqpC* in pneumococcal damage to macrophages was examined. Upon bacterial challenging, only 30% of macrophage cells died from coincubation with the ΔPn*-aqpC* mutant, compared to 72% and 65% cell death from coincubation with the wild-type and Pn*-aqpC*-com strains, respectively (Fig. 5B and C), whereas the addition of 1 KU/ml catalase reduced macrophage damage from the wild type by 13% (59% with versus 72% without catalase) but did not alleviate the damage from the ΔPn*-aqpC* mutant (Fig. 5B and C), indicating that Pn-AqpC-promoted H_2_O_2_ production has some contributions to macrophage death. The roles of Pn-AqpC and H_2_O_2_ in damage to macrophages were also verified by the activities of lactate dehydrogenase in the cultures leaked from macrophages (Fig. 5D). However, the catalase-treated wild-type cells still caused significantly higher macrophage death (59%) than the ΔPn*-aqpC* mutant (30%), implying that Pn-AqpC itself or other Pn-AqpC-impacted factors, possibly the reduced release of Ply, contribute to macrophages death.

### Pn-AqpC is required for pneumococcal virulence in a murine pulmonary infection model.

Given that H_2_O_2_ and Ply are major virulence factors (1, 2), and the deletion of Pn*-aqpC* not only increased the H_2_O_2_ and NO susceptibility of but also reduced Ply release by pneumococci, the contributions of Pn-AqpC to pneumococcal virulence were evaluated in a murine pneumonia infection model. BALB/c mice were intratracheally infected with 1.0 × 10^7^ CFU of D39, its Pn*-aqpC* deletion mutant (pretreated with or without 40 μM H_2_O_2_), and the Pn*-aqpC*-com strain. By monitoring mouse survival for 12 days postinfection, we found that the Pn-*aqpC* deletion significantly enhanced mouse survival to 78%, compared with 22% and 43% survival rates in the wild-type- and Pn*-aqpC*-com-infected groups, respectively (Fig. 5E), indicating that Pn-AqpC is involved in pneumococcal pathogenicity. Of note, the survival rate of mice infected with the 40 μM H_2_O_2_-pretreated ΔPn-*aqpC* mutant was reduced to 60%, compared with 78% survival of those infected by the non-H_2_O_2_-pretreated ΔPn-*aqpC* strain, suggesting that low-H_2_O_2_-induced oxidative resistance enhances pneumococcal pathogenicity in addition to other virulence factors.

Neither was inflammatory cell immersion (Fig. 5F) observed nor were pneumococci recovered from the lung tissue of the surviving mice infected by the ΔPn*-aqpC* mutant, whereas 1.35 × 10^8^ ± 0.91 × 10^8^ and 1.54 × 10^8^ ± 0.72 × 10^8^ CFU/ml of pneumococci were recovered from the lungs of dead mice infected by the D39 wild-type and Pn*-aqpC*-com strains, respectively. These data confirmed the contribution of Pn-AqpC to pneumococcal pathogenicity.

## DISCUSSION

**FIG 6:**
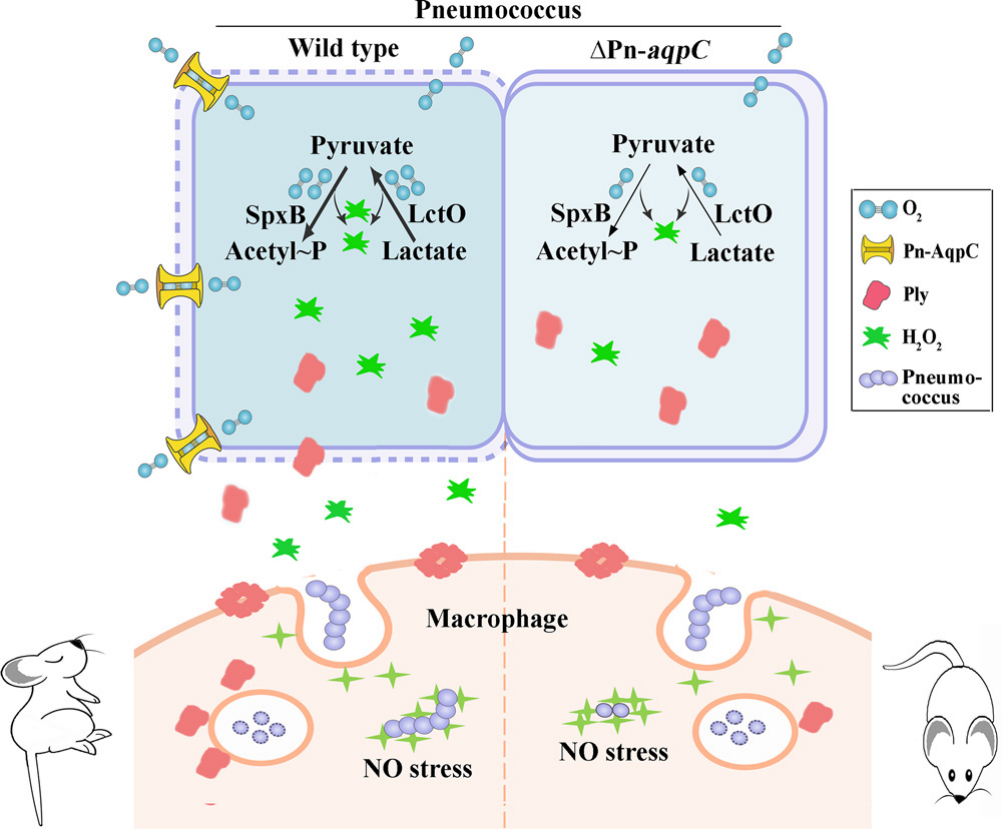
The newly identified oxygen-facilitating aquaporin Pn-AqpC modulates H_2_O_2_ production, ROS and RNS resistance, and pneumolysin (Ply) release and contributes significantly to the pathogenicity of pneumococcus. Pn-AqpC, an atypical aquaglyceroporin, functions as an oxygen porin to facilitate O_2_ influx and promotes pneumococcus to produce H_2_O_2_ via pyruvate oxidase (SpxB) and lactate oxidase (LctO). Endogenous H_2_O_2_ endows but deletion of Pn-*aqpC* reduces pneumococcus resistance to higher exogenous H_2_O_2_ and NO levels; therefore, the presence of Pn-AqpC enhances the survival of pneumococci in macrophages. Additionally, the presence of Pn-AqpC promotes pneumococcal cell lysis and, thus, Ply release. As the pneumococcal hemolysin Ply perforates eukaryotic cellular membranes and induces macrophage necroptosis, the presence of Pn-AqpC enhances pneumococcal damage to macrophages. Consistently, the absence of Pn-AqpC significantly attenuates the virulence of S. pneumoniae in a murine pneumonia model.

To date, 13 and 120 aquaporin isoforms have been identified in humans and plants, respectively, and are delineated into three major subfamilies: the classical water-transporting aquaporins, glycerol-transporting aquaglyceroporins, and AQP supergene channel superaquaporins (14, 15, 34). They facilitate the transmembrane diffusion of water, glycerol, H_2_O_2_, CO_2_, and other small uncharged solutes (14–16, 26, 34, 35). Here, we report a new aquaporin subfamily represented by pneumococcal Pn-AqpC, which functions as an oxygen porin to facilitate oxygen uptake. Phylogenetically, the oxygen porins are distantly related to aquaglyceroporins and possess substrate-selective amino acid residues distinct from those of aquaporins and aquaglyceroporins. Importantly, the oxygen porin Pn-AqpC contributes significantly to the pathogenicity of S. pneumoniae. As depicted in Fig. 6, pneumococcal Pn-AqpC, which is increasingly synthesized under conditions of higher O_2_ contents, facilitates O_2_ influx into cells and thus promotes H_2_O_2_ production by pneumococcus. Endogenous H_2_O_2_ helps pneumococci adapt to higher exogenous H_2_O_2_ and NO levels; therefore, the deletion of Pn-*aqpC* reduced the H_2_O_2_ and NO resistance of pneumococci. Accordingly, the presence of Pn-AqpC promotes pneumococcal survival in macrophages and possibly other host immune cells. In addition, the absence of Pn-AqpC alleviates pneumococcal autolysis and, thus, Ply release and significantly reduces pneumococcal damage to macrophages. In support of this, the absence of Pn-AqpC significantly attenuated the virulence of pneumococcus in a murine pneumonia model. Thus, the new subfamily of prokaryotic aquaporins, represented by Pn-AqpC, might be virulence-related proteins.

To our knowledge, Pn-AqpC is the first reported oxygen porin with defined physiological functions. Although O_2_ freely diffuses across the cytoplasmic membrane (36), assays of both *in vivo* and heterogeneously expressed yeast and *in vitro*-reconstituted proteoliposomes all determined that Pn-AqpC increases O_2_ flux across the cellular membrane (Fig. 2), particularly when pneumococci are grown under lower O_2_ levels (Fig. 2J and K) similar to those in most host environments (11). In addition, the pneumococcal cellular membrane appears to have lower O_2_ permeability than that of E. coli, highlighting the role of Pn-AqpC in pneumococci, which could require controllable O_2_ influx for H_2_O_2_ synthesis. Enhanced Pn-AqpC contents were found in aerobically grown pneumococcus (Fig. 3), conforming to its oxygen facilitator mission; however, similar Pn-*aqpC* transcript levels were found in aerobic and anaerobic cultures (data not shown), implying the posttranscriptional regulation of Pn-AqpC expression. So far, an O_2_-facilitating function has been reported only for human AQP1 and Nicotiana tabacum PIP1;3 when ectopically expressed in yeast (26). They are affiliated with water-type aquaporins and distantly related to Pn-AqpC at very low protein identities (20% and 14%, respectively) and distinct selective filter residues; therefore, prokaryotic Pn-AqpC represents a novel subfamily of aquaporins. Analogous to other aquaporin orthologs, Pn-AqpC forms a tetramer, as indicated by structural homology modeling implemented in SWISS-MODEL (Fig. S2A) and a macromolecular aggregate formed by the purified Pn-AqpC–10×His protein (Fig. 2F and G). Although O_2_ is predicted to permeate through the central pore of the four monomers of human AQP1 (36), Pro232 in the Pn-AqpC ar/R region has been determined to be crucial for facilitating O_2_ transport (Fig. 2B and C). Thus, O_2_ could be transported through the oxygen porin substrate channel in addition to the tetramer central pore. Thus far, only O_2_, but neither H_2_O nor glycerol and H_2_O_2_, has been verified as the substrate of Pn-AqpC (Fig. 2; see also Fig. S3 in the supplemental material). Of note, distinct from most other reported aquaporins, Pn-AqpC does not contain cysteine residues and thus is not inactivated by mercury chloride (data not shown).

Significantly, the oxygen porin Pn-AqpC contributes to pneumococcal pathogenicity, as the deletion of Pn-*aqpC* markedly attenuated lethality to mice (Fig. 5E). Through an exhaustive search, Pn*-aqpC* orthologs were found in all 8,183 pneumococcal genomes and contigs, which are attributed to 77 capsular serotypes and capsule-free strains. These orthologs exhibit 97% to 100% amino acid sequence identities with Pn-AqpC and 100% identity of YVPR in the ar/R region (Fig. 1C; Data Set S1). Additionally, Pn-AqpC orthologs are widely distributed among members of the genera Streptococcus and *Lactococcus* of the *Streptococcaceae* family, the genera *Lactobacillus* and *Pediococcus* of the *Lactobacillaceae* family, the genera *Oenococcus* and *Weissella* of the *Leuconostocaceae* family, and the genus *Enterococcus* of the *Enterococcaceae* family (Fig. 1C), so the members of this aquaporin subfamily appear to be restrictively present in facultative anaerobic bacteria, implying that they could contribute to the oxidative adaptation of these bacteria through O_2_ influx-enabled endogenous H_2_O_2_ production. Of note, Pn-AqpC orthologs are particularly prominent in some pathogenic streptococcal species, such as Streptococcus pyogenes and S. mutans (Fig. 1A), implying their association with virulence. Although the non-H_2_O_2_-producing species S. mutans also possesses a Pn-AqpC ortholog (SMU_396), its O_2_-facilitating role is not likely involved in H_2_O_2_ production through oxidases, whereas S. mutans encodes H_2_O-forming NADH oxidase (Nox), which uses O_2_ to oxidize NADH to NAD^+^ to achieve cellular redox balance and energy production (37). Deletion of the *nox* gene reduced O_2_ consumption by pneumococcus. A previous study also found that O_2_ promotes O_2_-tolerant S. mutans growth (38); thus, S. mutans AqpC could also have an important physiological role.

10.1128/mBio.01309-21.10 DATA SET S1 (Sheet 1) Pn-*aqpC* (Spr1344) of pneumococcus R6 hits for aquaporin orthologs in 8,183 completed genomes and contig sequences of the pneumococcal strains attributed to 77 different pneumococcal serotypes and nondefined serotypes by performing BLASTP analysis. (Sheet 2) Sequences representative of various pneumococcus serotypes possessing Pn-AqpC homologs. (Sheet 3) Amino acid sequences of Pn-AqpC homologs in 8,183 pneumococcus sequenced genomes and contigs. Download Data Set S1, XLSX file, 2.0 MB. Copyright © 2021 Hu et al. 2021 Hu et al. https://creativecommons.org/licenses/by/4.0/ This content is distributed under the terms of the Creative Commons Attribution 4.0 International license.

Based on the experimental evidence of pneumococcal survival in and damage to macrophages (Fig. 5A to D), we hypothesize that the mechanistic basis of Pn-AqpC in pneumococcal pathogenicity lies in its control of pneumococcal H_2_O_2_ production and Ply release. Compared with other pathogenic bacteria, streptococci are highly capable of resisting oxidative stress via endogenous H_2_O_2_-induced resistance to higher levels of exogenous H_2_O_2_ (6, 39), and this unique characteristic enables them to defend against the innate immune system of the infected host (9). H_2_O_2_ also contributes to pneumococcal virulence by damaging alveolar epithelial cell DNA and suppressing host innate immune systems (4, 5). Consistently, the major H_2_O_2_-producing enzyme pyruvate oxidase is crucial for the virulence of S. pneumoniae (40). This work identified that Pn-AqpC, by facilitating O_2_ uptake, acts as a novel key component in controlling H_2_O_2_ production and oxidative stress resistance; thus, the absence of this membrane protein causes S. pneumoniae to be rapidly eliminated by macrophages and reduces damage to macrophages. Survival in macrophages could be important for pneumococcal invasion and is critical for pneumococcal bacteremia and persistence within hosts (10, 41). Remarkably, the presence of Pn-AqpC elevates pneumococcal autolysis and Ply release (Fig. 4E and F), probably due to endogenous H_2_O_2_ production, thus increasing the hemolytic activity of pneumococci. Ply, as the major virulence factor of pneumococci, has been known to mediate bacterial transmission, trigger inflammatory responses, and cause macrophage necrosis (3, 42, 43). Rapid autolysis and pneumolysin release were reported to increase the pathogenicity of pneumococcal serotype 1 (44). In addition, no correlations have been found between Pn-AqpC and other identified pneumococcal virulence factors, as the deletion of Pn*-aqpC* did not alter the transcription of *lytA*, *psaA*, *pspC*, *spxB*, and *lctO* (Fig. S4) or the capsule polysaccharide amounts (Fig. S6). Therefore, the virulence relevance of Pn-AqpC lies mainly in its oxygen-transporting function.

10.1128/mBio.01309-21.7 FIG S6 Capsular polysaccharide (CPS) amounts were measured with a Stains-all assay. The D39 wild type (WT) and its ΔPn-*aqpC* and Pn-*aqpC*-complemented strains (Pn-*aqpC*-com) were grown on blood agar plates, collected, and resuspended in 150 mM Tris-HCl (pH 7.0)–1 mM MgSO_4_ to make the cell suspension to an OD_600_ of 3.5. An aliquot of 1 ml was centrifuged at 13,000 rpm for 10 min, and the pellet was resuspended in 0.5 ml of 150 mM Tris-HCl (pH 7.0)–1 mM MgSO_4_. The pellet suspension was supplemented with 0.1% (wt/vol) deoxycholate and incubated at 37°C for 15 min to induce cell autolysis; 100 U of mutanolysin, 50 μg of DNase I, and 50 μg of RNase A were then added; and the mixture was incubated for 18 h. The samples were then incubated with 50 μg of proteinase K at 56°C for 4 h. Next, all the samples were 5-fold diluted with 200 μl 150 mM Tris-HCl (pH 7.0), and CPS amounts in each sample were then determined by mixing 250 μl 5-fold-diluted samples with 1 ml of Stains-all solution containing 20 mg of 1-ethyl-2-{3-[1-ethylnaphtho-(1,2-*d*)thiazolin-2-ylidene]-2-methylpropenyl}naphtho-(1,2-*d*)thiazolium bromide (Sigma) and 60 μl of glacial acetic acid in 100 ml of 50% formamide and measuring the absorbance at 640 nm. The R6 wild-type strain was included as a background control. All experiments were conducted three times, and the averages ± SD from one independent assay on triplicate samples are shown. Download FIG S6, TIF file, 0.8 MB. Copyright © 2021 Hu et al. 2021 Hu et al. https://creativecommons.org/licenses/by/4.0/ This content is distributed under the terms of the Creative Commons Attribution 4.0 International license.

Collectively, Pn-AqpC, by facilitating O_2_ uptake, modulates H_2_O_2_ production and Ply release, the two major virulence factors of pneumococci, and contributes remarkably to pneumococcal virulence. Pn*-aqpC* orthologs were found in all 8,183 pneumococcal genomes and contigs (Data Set S1). Therefore, the conserved membrane-integrated Pn-AqpC is exposed as a new potential target for fighting against pneumococcal disease.

## MATERIALS AND METHODS

### Experimental strains and growth.

Experimental strains are listed in Table S2 in the supplemental material. Pneumococcus was grown in brain heart infusion (BHI) broth or agar plates (BD Difco) with 5% sterile defibrinated sheep blood at 37°C with 5% CO_2_. Pneumococcal strains were grown statically, with shaking, or anaerobically under 100% nitrogen. When required, kanamycin (1 mg/ml) and spectinomycin (300 μg/ml) were added.

10.1128/mBio.01309-21.9 TABLE S2 Strains, plasmids, and primers used in this study. Download Table S2, DOCX file, 0.03 MB. Copyright © 2021 Hu et al. 2021 Hu et al. https://creativecommons.org/licenses/by/4.0/ This content is distributed under the terms of the Creative Commons Attribution 4.0 International license.

### Construction of genetically modified strains.

All primers are listed in Table S2. The PCR ligation method (45) was used to construct the Pn-*aqpC* and *nox* deletion strains and His-, photoactivatable fluorescent protein mMaple3 (29)-, or super folder green fluorescent protein (sfGFP)-tagged strains of S. pneumoniae. The spectinomycin and kanamycin resistance genes were derived from plasmids pDL278 (46) and pALH124 (47), respectively. The Pn-*aqpC* gene with its promoter was cloned into pDL278 for complemented strain construction. Alanine substitutions for Tyr49, Val223, Pro232, and Arg238 were implemented on pDL278-Pn-*aqpC* using a site-directed gene mutagenesis kit (Beyotime, China). Transformation was performed as described previously (48). Correct transformants were confirmed by PCR and DNA sequencing.

### Test of the transportable substrates of Pn-AqpC-constituted proteoliposomes.

The purified 10×His-tagged Pn-AqpC protein was reconstituted into liposomes made by the S. pneumoniae cellular membrane lipid (49, 50) and E. coli total lipid extract (Avanti) as previously described (16, 27). Detailed procedures are available in Text S1 in the supplemental material.

10.1128/mBio.01309-21.1 TEXT S1 Supplemental experimental procedures. Download Text S1, DOCX file, 0.04 MB. Copyright © 2021 Hu et al. 2021 Hu et al. https://creativecommons.org/licenses/by/4.0/ This content is distributed under the terms of the Creative Commons Attribution 4.0 International license.

To examine the O_2_ permeability of Pn-AqpC, cell membrane-impermeable and oxygen-quenchable phosphorescent oxygen probes (Cayman Chemical) were encapsulated into proteoliposomes and Pn-AqpC-devoid liposomes. The proteoliposomes and liposomes were vacuumed and N_2_ gas flushed for 7 cycles, and 100 μl per well was then dispersed into a 96-well plate (Corning) under air. The fluorescence intensity of the phosphorescent oxygen probe was monitored (excitation at 380 nm and emission at 650 nm) for a recommended delay of 30 μs using a Synergy H4 hybrid multimode microplate reader (BioTek). Water and glycerol permeabilities were assayed using an SX20 stopped-flow spectrometer as previously described (27, 51). The experiments were repeated three times.

### PALM imaging.

Mid-exponential-phase Pn*-aqpC*-mMaple3 cells were exposed to air for 30 min in the dark and then observed using PALM imaging (28) as previously described (12). The superresolution images were constructed using Insight3 software (52), which was kindly provided by Bo Huang (University of California, San Francisco). PALM data analyses such as drift correction, protein abundance, and image rendering were carried out using custom-written Matlab scripts.

### Assay of pneumococcal survival in macrophages and damage to macrophages.

Mouse monocyte-macrophage RAW 264.7 cells (1 × 10^5^) were challenged for 1 h with pneumococcus at a multiplicity of infection of 100:1. After removing the bacteria, macrophages were incubated for another 1 and 1.5 h to count CFU of pneumococci within macrophages or for 16 h to determine macrophage death. Detailed procedures are available in Text S1 in the supplemental material.

### *In vivo* mouse infection experiment.

BALB/c mice (specific-pathogen-free [SPF] grade) were purchased from Vital River Company (Beijing, China). Animal experiments were approved by the Biomedical Research Ethics Committee of the Institute of Microbiology, Chinese Academy of Sciences. The protocol was approved by the Institutional Animal Care and Use Committee. S. pneumoniae D39 and derivative strains were intratracheally administered to 6- to 8-week-old female BALB/c mice at 1 × 10^7^ CFU in 20 μl phosphate-buffered saline (PBS), and PBS-administered BALB/c mice were included as controls. Mouse survival (10 per group) was monitored for 12 days. Mice were sacrificed under anesthesia, half-lungs were ground for enumerating pneumococcal CFU, and the other halves were used for histopathological observation.

### Statistical analysis.

One-way analysis of variance (ANOVA) followed by Tukey’s *post hoc* test and Student’s *t* test was performed using PASW Statistics 18 and Excel, respectively. A log rank Mantel-Cox test was performed using GraphPad Prism 8.0. The level of significance was determined at a *P* value of <0.05.

### Other procedures.

Detailed procedures are available in Text S1 in the supplemental material.
