# Supplementary figures and images for: A Novel Aquaporin Subfamily Imports Oxygen and Contributes to Pneumococcal Virulence by Controlling the Production and Release of Virulence Factors
Source: mBio. 2021 Aug 17;12(4):e01309-21. doi: 10.1128/mBio.01309-21 (PMC8406300; doi:10.1128/mBio.01309-21)

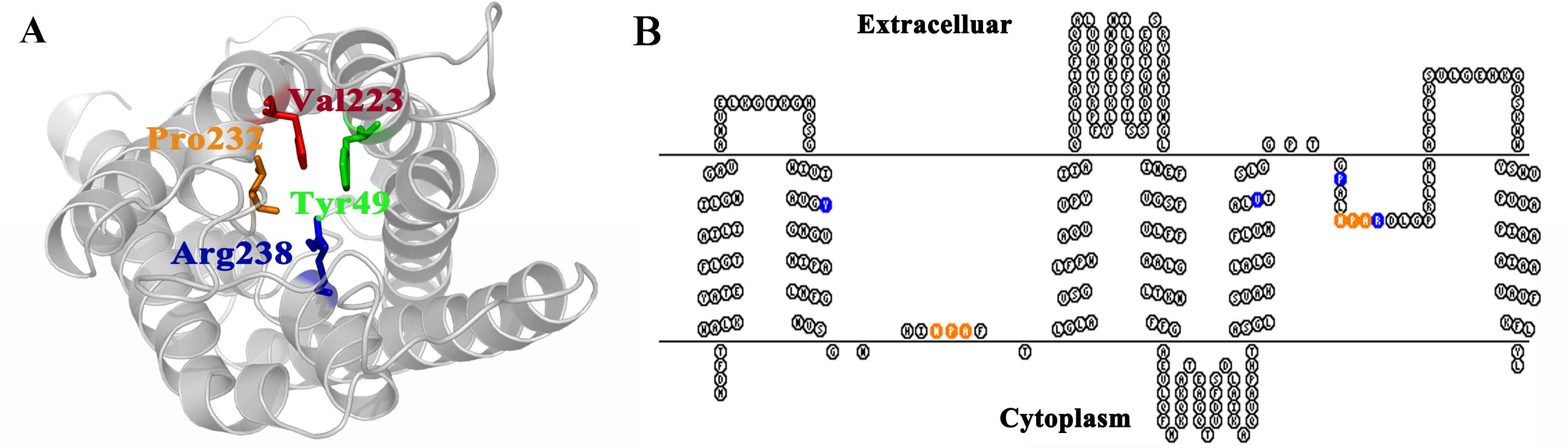

Supplement: FIG S1 [file mbio.01309-21-sf001.tif]

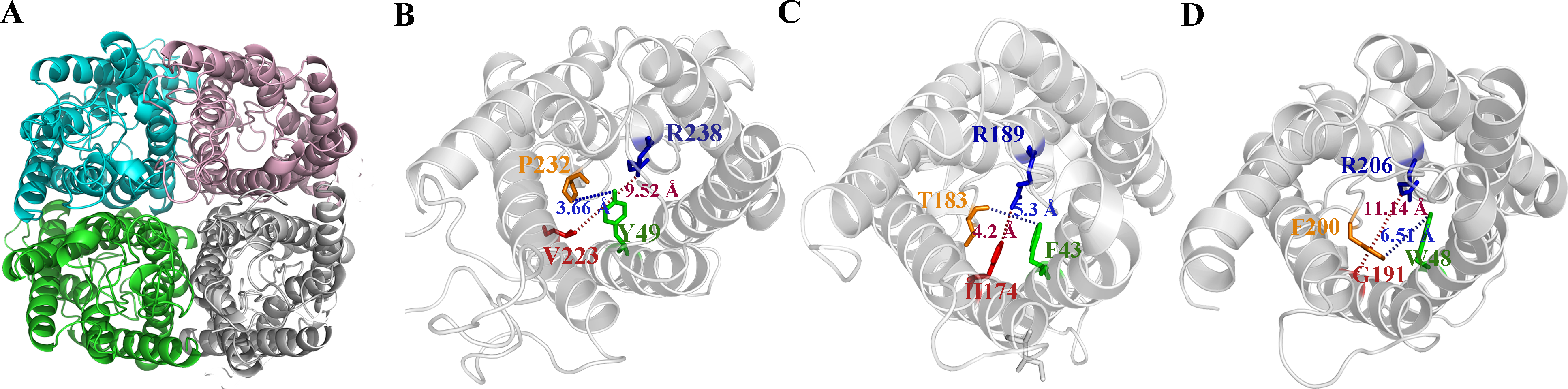

Supplement: FIG S2 [file mbio.01309-21-sf002.tif]

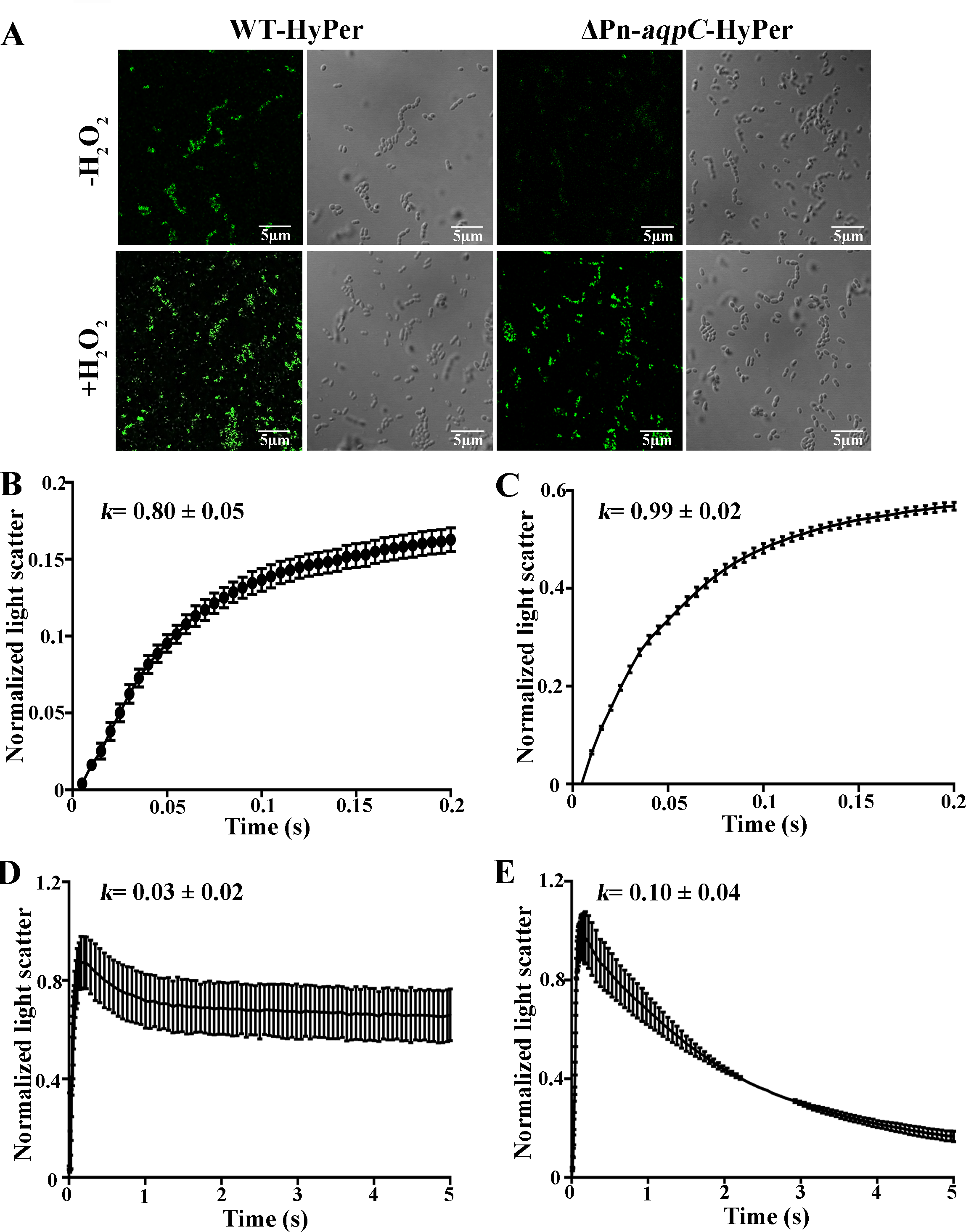

Supplement: FIG S3 [file mbio.01309-21-sf003.tif]

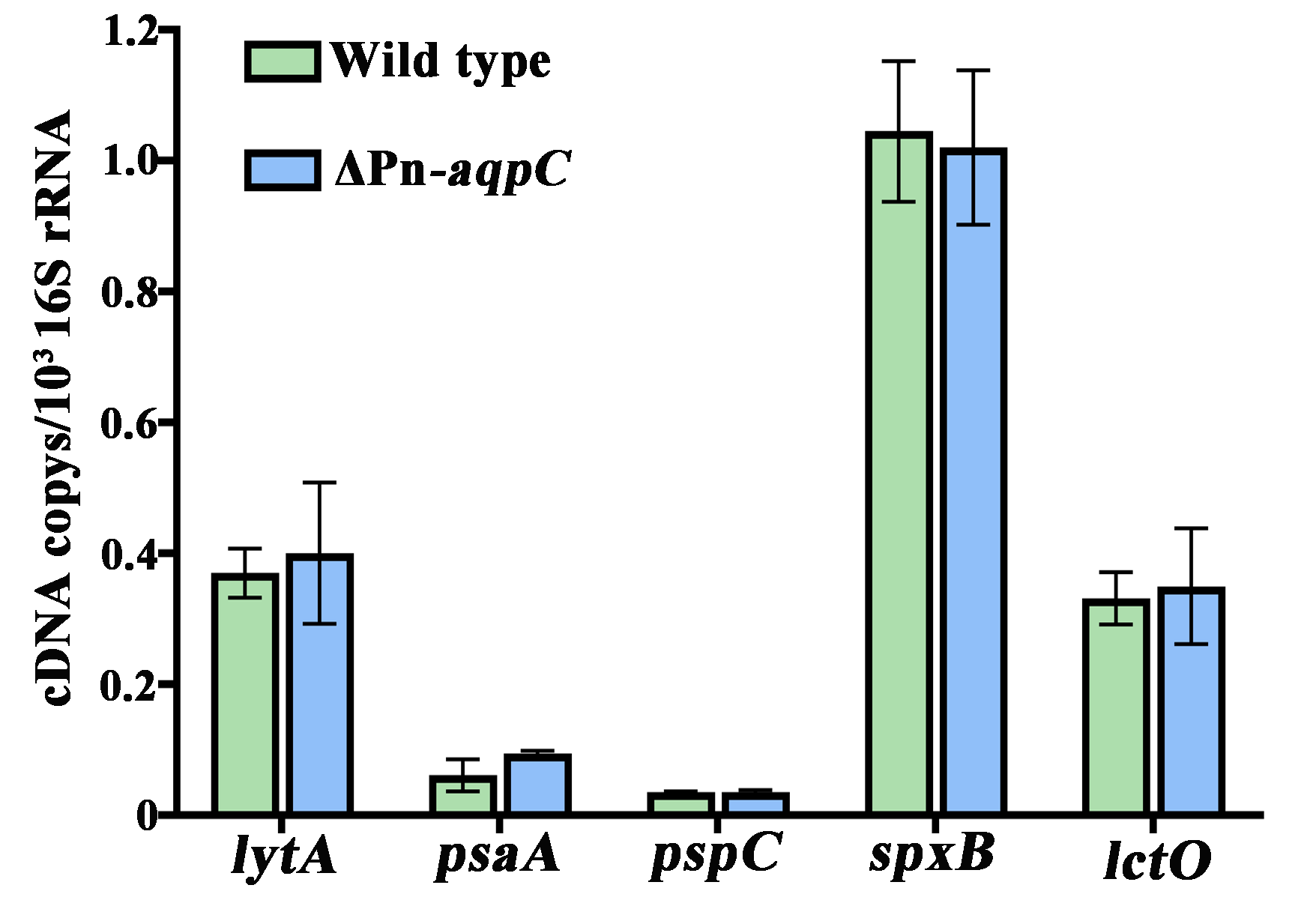

Supplement: FIG S4 [file mbio.01309-21-sf004.tif]

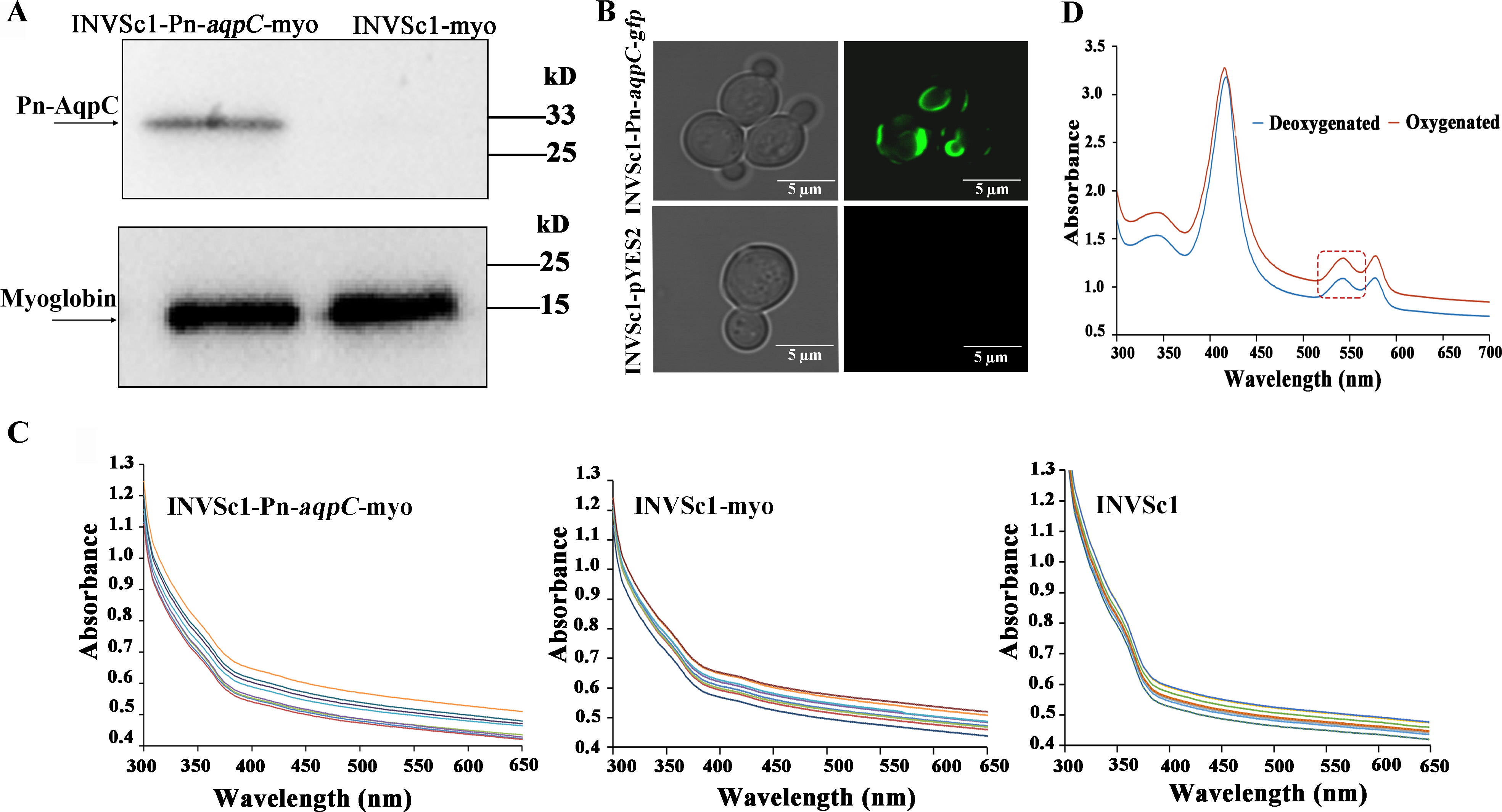

Supplement: FIG S5 [file mbio.01309-21-sf005.tif]

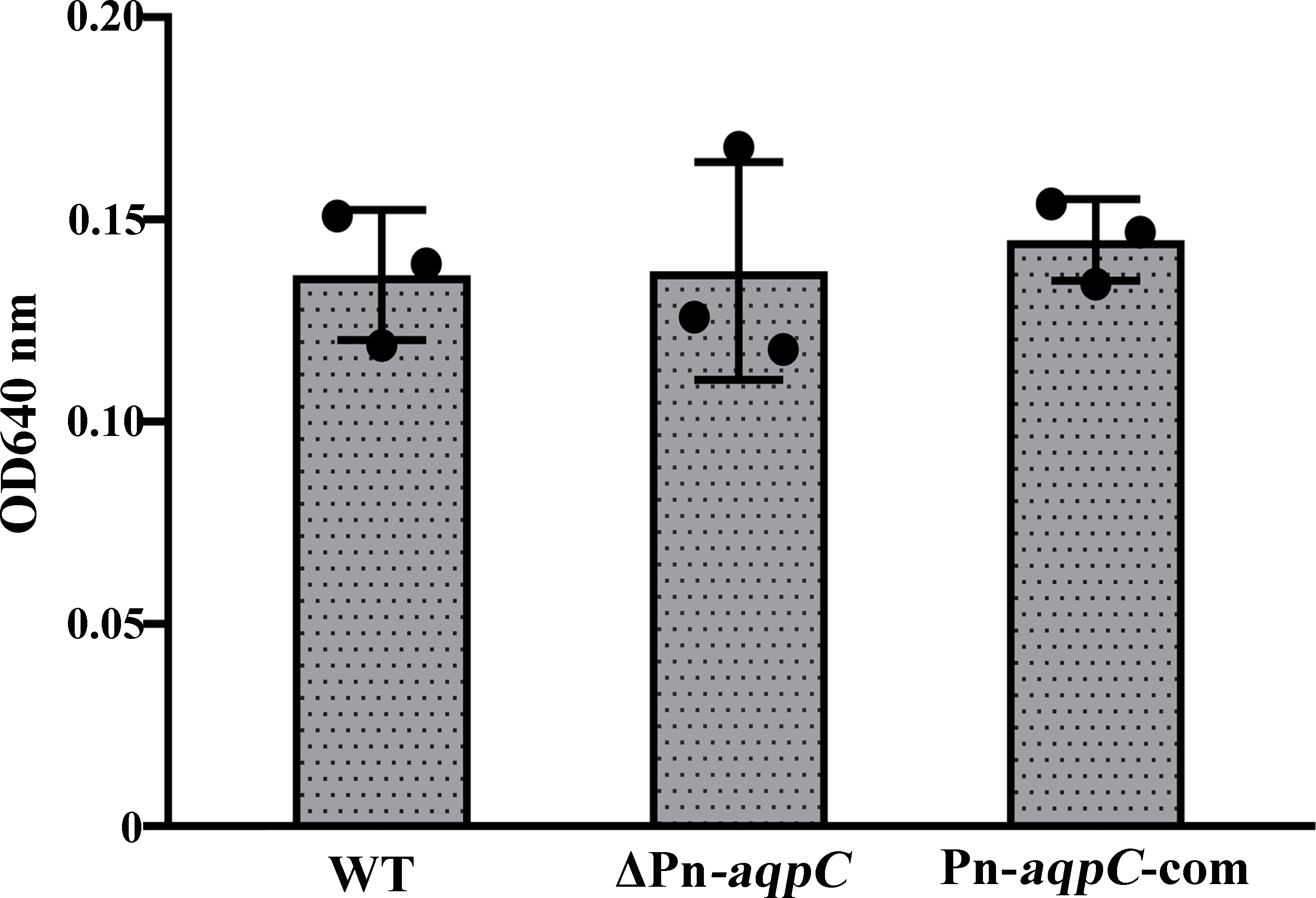

Supplement: FIG S6 [file mbio.01309-21-sf006.tif]
