# Supplementary material for: A Novel Aquaporin Subfamily Imports Oxygen and Contributes to Pneumococcal Virulence by Controlling the Production and Release of Virulence Factors
Source: mBio. 2021 Aug 17;12(4):e01309-21. doi: 10.1128/mBio.01309-21 (PMC8406300; doi:10.1128/mBio.01309-21)
Supplement: TABLE S1 [file mbio.01309-21-st001.docx]

**Table S1. LC/MS-MS identification of Pn-AqpC-10His proteins**

| Protein | Protein accession number | Coverage (%) | Matched peptides |
| --- | --- | --- | --- |
| Molecular weight at ~32 kD | Q8DP55 | 37 | KTENPNNILGTF  STISSIDHGTKESRY  MKQKATEAGQTVDFSDL  SVAHLALGF  HAFLPKSVLGEHKGDSKW |
| Molecular weight at ~68 kD | Q8DP55 | 57 | GNVSGNHINPAFTL  KTENPNNIL  STISSIDHGTKESRY  TKNFFGAEVL  MKQKATEAGQTVDFSDL  AIKAQVAPHTASGL  GGPTGPALNPARDL  NPARDLGPRLL  HAFLPKSVL  GEHKGDSKWWY |
