# Supplementary material for: A Novel Aquaporin Subfamily Imports Oxygen and Contributes to Pneumococcal Virulence by Controlling the Production and Release of Virulence Factors
Source: mBio. 2021 Aug 17;12(4):e01309-21. doi: 10.1128/mBio.01309-21 (PMC8406300; doi:10.1128/mBio.01309-21)
Supplement: TABLE S2 [file mbio.01309-21-st002.docx]

**Table S2. Strains, plasmids and primers used in this study^*^**

| Strain, plasmid or primer | Characteristics and description | Reference^a^ or source |
| --- | --- | --- |
| Strain |  |  |
| *E. coli* |  |  |
| DH5α | *supE44 lacU169* (80 *lacZ*M15) *hsdR17 recA1 endA1 gyrA96 thi-1 relA1 luxS* | TransGen Biotech, Beijing, China |
| BL21 (DE3) | F- *ompT* *hsdS* (rBB - mB -) gal dcm (DE3) | TransGen Biotech, Beijing, China |
| *S. pneumoniae* |  |  |
| D39 Wild-type | D39Kan^s^ Sp^s^ | 1 |
| D39 ∆Pn-*aqpC* | *aqpC*::Kan; Kan^r^; D39 with *aqpC* deletion | This study |
| D39 Pn-*aqpC-*com | D39*aqpC*::Kan; pDL278-Pn-*aqpC*; Kan^r^; Sp^r^; ∆Pn-*aqpC* with *aqpC* ectopically expressed | This study |
| R6 Wild-type | R6 Kan^s^ Sp^s^ | ATCC, Manassas, USA |
| R6 ∆Pn-*aqpC* | R6*aqpC*::Kan; Kan^r^; R6 with *aqpC* deletion | This study |
| R6 Pn-*aqpC-*com | R6*aqpC*::Kan; pDL278-Pn-*aqpC*; Kan^r^; Sp^r^; ∆Pn-*aqpC* with *aqpC* ectopically expressed | This study |
| WT-HyPer | R6 harboring pDL278-P*ldh*-HyPer; Sp^r^; R6 with HyPer gene ectopically expressed | This study |
| ∆Pn-*aqpC-*HyPer | R6*aqpC*::Kan; pDL278-P*ldh*-HyPer; Kan^r^ Sp^r^; ∆Pn-*aqpC* with HyPer gene ectopically expressed | This study |
| Tyr49A | R6 *aqpC*::Kan; pDL278-Pn-*aqpC*Tyr49A; Kan^r^; Sp^r^; ∆Pn-*aqpC* with *aqpC*Tyr49A ectopically expressed | This study |
| Val223A | R6 *aqpC*::Kan; pDL278-Pn-*aqpC*Val223A; Kan^r^; Sp^r^; ∆Pn-*aqpC* with *aqpC*Val223A ectopically expressed | This study |
| Pro232A | R6 *aqpC*::Kan; pDL278-Pn-*aqpC*Pro232A; Kan^r^; Sp^r^; ∆Pn-*aqpC* with *aqpC*Pro232A ectopically expressed | This study |
| Arg238A | R6 *aqpC*::Kan; pDL278-Pn-*aqpC*Arg238A; Kan^r^; Sp^r^; ∆Pn-*aqpC* with *aqpC*Arg238A ectopically expressed | This study |
| WT-pDL278 | R6 carrying pDL278; Kan^s^ Sp^s^; | This study |
| ∆Pn-*aqpC-*pDL278 | R6*aqpC*::KanpDL278; Kan^r^; R6 with *aqpC* deletion and carrying pDL278 | This study |
| Ply-6His | R6 *ply*::6His; Sp^r^; R6 with *ply* fused with 6×His tag at C-terminus | This study |
| ∆Pn*-aqpC*-Ply-6His | R6 *aqpC*::Kan *ply*::6His; Kan^r^; Sp^r^; ∆Pn-*aqpC* with *ply* fused with 6×His tag at C-terminus | This study |
| Pn-*aqpC*-mMaple3 | R6*aqpC*::mMaple3; Sp^r^; R6 with *aqpC*-mMaple3 fusion | This study |
| Pn-*aqpC*-*gfp* | R6*aqpC*::sf*gfp*; Sp^r^; R6 with *aqpC*-sfGFP fusion | This study |
| *Saccharomyces cerevisiae* |  |  |
| INVSc1 | INVSc1 MATa his3∆1 leu2 trp1-289 ura3-52/ MATα his3∆1 leu2 trp1-289 ura3-52; His^-^, Leu^-^, Trp^-^, Ura^-^; Kan^r^; Amp^r^ | Invitrogen, Carlsbad, USA |
| INVSc1-pYES2 | INVSc1 harboring pYES2 | This study |
| INVSc1-Pn-*aqpC*-*gfp* | INVSc1 harboring pYES2-Pn-*aqpC-gfp* | This study |
| INVSc1-Pn-*aqpC-*myo | INVSc1 harboring pAG425GAL-ccdB-myo and pAG426GAL-ccdB-Pn-*aqpC*; Kan^r^; Amp^r^ | This study |
| INVSc1-myo | INVSc1 harboring pAG425GAL-ccdB-myo and pAG426GAL-ccdB; Kan^r^; Amp^r^ | This study |
| **Plasmid** |  |  |
| pGEX-4T-1 | Amp^r^ | GE Healthcare, MA, USA |
|  |  |  |
| pGEX4T-Pn-AqpC | Amp^r^ | This study |
| pMB413a | pUC19 carrying Sperm Whale Myoglobin gene | Addgene, MA, USA |
| pAG425GAL-ccdB | Cat^r^; Amp^r^; Selectable markers LEU2; GAL promoter | Addgene, MA, USA |
| pAG426GAL-ccdB | Cat^r^; Amp^r^; Selectable markers URA3; GAL promoter | Addgene, MA, USA |
| pAG425GAL-ccdB -myo | pAG425GAL-ccdB carrying myoglobin-6×His | This study |
| pAG426GAL-ccdB-Pn-*aqpC* | pAG425GAL-ccdB carrying Pn*-aqpC*-3×Flag | This study |
| pYES2 | Cat^r^; Amp^r^; Selectable markers URA3; GAL promoter | Invitrogen, Carlsbad, USA |
| pYES2-*So*-*aqpA*-*gfp* | pYES2 carrying *So*-*aqpA*-*gfp* | 2 |
| pYES2-Pn-*aqpC-gfp* | pYES2 carrying Pn*-aqpC*-*gfp* | This study |
| pALH124 | Kan^r^ | 3 |
| pDL278 | Sp^r^ | 4 |
| pDL278-Pn-*aqpC* | pDL278 carrying Pn-*aqpC*; Sp^r^ | This study |
| pDL278-Pn-*aqpC*Tyr49A | pDL278 carrying Pn-*aqpC*Tyr49A; Sp^r^ | This study |
| pDL278-Pn-aqpCVal223A | pDL278 carrying Pn-aqpCVal223A; Sp^r^ | This study |
| pDL278-Pn*-aqpC*Pro232A | pDL278 carrying Pn*-aqpC*Pro232A; Sp^r^ | This study |
| pDL278-Pn*-aqpC*Arg238A | pDL278 carrying Pn*-aqpC*Arg238A; Sp^r^ | This study |
| pDL278-HyPer | pDL278 carrying HyPer gene; Sp^r^ | 2 |
| pDL278-P*ldh*-HyPer | pDL278-HyPerintegratingpromoter of lactate dehydrogenase; Sp^r^ | This study |
| **Primer** | **Sequence (5’-3’)** | **Purpose** |
| *aqpC*F | GATGTTGTTCAACCTGTAACCTTAAGCCG | *aqpC* deletion |
| *aqpC*upRBamHI | CGG*GGATCC*CATTTGCTAGATTCTCCTTAAAATTTTTTA GATATTCC | *aqpC* deletion |
| *aqpC*dnFBamHI | CGG*GGATCC*TAAGAAATAGCTCCTTTAACATTTGAGTGAG | *aqpC* deletion |
| *aqpC*dnR | GAAAGTACTAAATAGACTTATCCTTCAAGG AG | *aqpC* deletion |
| *aqpC*-comF | CGGGATCCTAGATTTTAGAATCTAGGAACC | Pn-*aqpC*-com |
| *aqpC*-comR | CTAATCGGTTAATGATGATGATGATGATGATGATG | Pn-*aqpC*-com |
| HyPerFBamHI | AGC*GGATCC*TAATAAATGCCTCATTTC | P*ldh*-HyPer fusion construction |
| HY-LDH | CTCGCCCTGCTGGCTTGCCATTTGTTTTAA ACATCTCCTTA | P*ldh*-HyPer fusion construction |
| LDH-HY | TAAGGAGATGTTTAAAACAAATGGCAAGCCAGCAGGGCGAG | P*ldh*-HyPer fusion construction |
| HyPerRHindIII | AAAA*AAGCTT*TTAAACCGCCTGTTTTAAAAC | P*ldh*-HyPer fusion construction |
| *aqpC*F | GGCGGCATCCAAATTCGGGTGTCG | Pn-*aqpC-gfp* strain |
| *aqpC*-*sfgfp*R | CGC*GGATCC*TCATTTGTACAGTTCATCCA TACCATG | Construction of Pn-*aqpC-gfp* strain |
| *sfgfp-aqpC*F | AT*GGATCC*AGAAACATCTTTTTCAACAAAATGC | Pn-*aqpC-gfp* strain |
| *sfgfp*BamHIR | GGCTGACGCCATTTCAAATGC | Pn-*aqpC-gfp* strain |
| *aqpC-*mMaple3R | CTTTAGAACCGCCGCCACCAGAGAGATAAAGGAATTTGAATACAG | Pn-*aqpC-* mMaple3 strain |
| mMaple3*-aqpC*F | CTGTATTCAAATTCCTTTATCTCTCTGGTGGCGGCGGTTCTAAAG | Pn-*aqpC* mMaple3 strain |
| mMaple3-BamHIR | CTAGGATCCTTATTTGTACAGCTCATCCATG | Pn-*aqpC* mMaple3 strain |
| Tyr49AF | GATCGTCATCGCTGTTGGTGCAGGTATGGGGGTTATGATC | Tyr49A |
| Tyr49AR | GATCATAACCCCCATACCTGCACCAACAGCGATGACGATC | Tyr49A |
| Val223AF | GATTCCTCGTTATGGCTTTGgcaACATCACTTGGAGGACCTAC | Val223A |
| Val223AR | GTAGGTCCTCCAAGTGATGTTGCCAAAGCCATAACGAGGAATC | Val223A |
| Pro232AF | CTTGGAGGACCTACAGGAgcaGCCTTGAACCCAGCCCG | Pro232A |
| Pro232AR | CGGGCTGGGTTCAAGGCTGCTCCTGTAGGT CCTCCAAG | Pro232A |
| Arg238F | CTGCCTTGAACCCAGCCGCAGACTTGGGACCACGTCTC | Arg238A |
| Arg238R | GAGACGTGGTCCCAAGTCTGCGGCTGGGTT CAAGGCAG | Arg238A |
| PlyHisF | GCAGATTTATTATACAGTCAGCGTAGACG | *Ply*-6His construction |
| PlyHisBamHIR | CGC*GGATCC*CTAATGGTGATGGTGATGATGGTCATTTTCTACCTTATCCTCTACC | *Ply*-6His construction |
| PlydownBamHIF | CGC*GGATCC*GAGAGGAGAATGCTTGCGACAAAAAGAGGCG | *Ply*-6His construction |
| PlydownR | GCAAATCAAGCTAGGATAGCACTTTACC | *Ply*-6His construction |
| Pn-AqpC-10HisEcoRIF | CG*GAATTC*ATGGATTTCACATGGGCACTG | Overexpression of Pn-AqpC-10His |
| Pn-AqpC-10HisXhoIR | CCG*CTCGAG*TTAATGATGATGATGATGATGATGATGATGATGGAGATAAAGGAATTTGAATACAGCTACTGC | Overexpression of Pn-AqpC-10His |
| pAG426-*aqpC*F | GACTAGTATGGATTACAAGGACGACGATGACAAGGATTACAAGGACGACGATGACAAGGATTACAAGGACGACGATGACAAGGCCACCATGGATTTCACATGGGC | Recombinant  3xFlag tagged AqpC |
| pAG426-*aqpC*R | GCAAGAATGCGGCCGCTTAGAGATAAAGGAATTTGAATACAG | Recombinant  3xFlag tagged AqpC |
| pAG425myoglobinF | GGACTAGTATGATGGTTCTGTCTGAAGGTG | Recombinant  Myoglobin-6His expression |
| pAG425myoglobinR | CCGAAGCTTTTAATGATGATGATGATGATGACCCTGGTAACCCAG | Recombinant  Myoglobin-6His expression |
| pYES2-*aqpC-sfgfp*F | CTATAGGGAATATTAAGCTTATGGATTTCACATGGGCACTGAAG | Recombinant *aqpC*-*gfp* expression |
| pYES2-*aqpC-sfgfp*R | AGCTCTTCGCCTTTACGCATGAGATAAAGGAATTTGAATACAGCTAC | Recombinant *aqpC*-*gfp* expression |
| *nox*UPF | GACAACAGGGATGGTCATTAAATGATTTC | *nox* deletion |
| *nox*BamHIUPR | CT*GGATCC*CATAAAAATTTCCTGCCTTCATTCTAAATTTAC | *nox* deletion |
| *nox*BamHIDnF | CT*GGATCC*TAAAAATGAATGAGCTATCTGGCCTTAAG | *nox* deletion |
| *nox*DnR | CTCCAGCAGCGAAATTTACAACTGG | *nox* deletion |
| 16SRTF | CGCATAAGAGTGGATGTTGCATGAC | 16S rDNA qPCR |
| 16SRTR | CTTCCGTCCA TTGCCGAAGA TTC | 16S rDNA qPCR |
| *pspA* RTF | CGAATACGAACCACTTCCTGAAGACG | *pspA* qPCR |
| *pspA* RTR | GTTAAGCCAA GCGTGTGGGT CTTC | *pspA* qPCR |
| *psaA* RTF | CGAATACGAACCACTTCCTGAAGACG | *psaA* qPCR |
| *psaA* RTR | GTTAAGCCAA GCGTGTGGGTCTTC | *psaA* qPCR |
| *lytA* RTF | CCATATAGGCAAGTACACGCACACTC | *lytA* qPCR |
| *lytA* RTR | GGTAGTCCGT CATGAACTCT TCTTTGG | *lytA* qPCR |
| *spxB* RTF | GCAGACACAGTTCTTTTCCTTGGTTC | *SpxB* qPCR |
| *spxB* RTR | CGTTTGCACGCCACCATGGAG | *SpxB* qPCR |
| *lctO* RTF | CAAGGGACCACAATGCCGTG | *lctO* qPCR |
| *lctO* RTR | GACCAATAGCTACCAAGTCTGCTC | *lctO* qPCR |

^*^: Amp, ampicillin; Kan, kanamycin; Sp, spectinomycin; Cat, Chloramphenicol; r, resistant; s, sensitive.

Italic nucleotide bases indicate restriction enzyme digestion sites.

^a^,**References:**

1. Li G, Liang Z, Wang X, Yang Y, Shao Z, Li M, Ma Y, Qu F, Morrison DA, Zhang JR. 2016. Addiction of hypertransformable pneumococcal isolates to natural transformation for in vivo fitness and virulence. Infect Immun 84:1887-1901.

2. Tong H, Wang X, Dong Y, Hu Q, Zhao Z, Zhu Y, Dong L, Bai F, Dong X. 2019. A *Streptococcus* aquaporin acts as peroxiporin for efflux of cellular hydrogen peroxide and alleviation of oxidative stress. J Biol Chem 294:4583-4595.

3. Liu Y, Zeng L, Burne RA. 2009. AguR is required for induction of the *Streptococcus mutans* agmatine deiminase system by low pH and agmatine. Appl Environ Microbiol 75:2629-2637.

4. Podbielski A, Spellerberg B, Woischnik M, Pohl B, Lutticken R. 1996. Novel series of plasmid vectors for gene inactivation and expression analysis in group A streptococci (GAS). Gene 177:137-147.
