# Supplementary material for: A Novel Aquaporin Subfamily Imports Oxygen and Contributes to Pneumococcal Virulence by Controlling the Production and Release of Virulence Factors
Source: mBio. 2021 Aug 17;12(4):e01309-21. doi: 10.1128/mBio.01309-21 (PMC8406300; doi:10.1128/mBio.01309-21)
Supplement: TEXT S1 [file mbio.01309-21-s0001.docx]

**Supplemental Experimental Procedures**

**Overexpression of Pn-AqpC-10His protein and reconstitution of Pn-AqpC into proteoliposomes.** The C-terminal 10His tagged R6 Pn-*aqpC* gene was cloned into plasmid pGEX-4T-1 (GE Healthcare) and transformed into *E. coli* BL21 (DE3) (TransGen Biotech, Beiijng, China). Then BL21-pGEX4T-Pn-AqpC cells of OD_600_ ~1.0 were added with final concentration of 0.1 mM of isopropyl-β-D-thiogalactopyranoside (IPTG, Sigma-Aldrich) and incubated at 22°C for overnight. The cells were lysed by sonication, and supernatants were further centrifuged at 100,000 g for 1 h. GST-Pn-AqpC-10His protein was solubilized from the cellular membrane using 2% N-Dodecyl-β-D-maltoside (DDM, Macklin, China) at 4°C for overnight. Then the supernatant was applied to a GSTrap HP column (GE Healthcare). Proteins were eluted by 20 mM Tris-HCl buffer (pH 8.0) containing 1% Octylglucoside (OG, Apexbio) and 10 mM reduced glutathione, and digested by 100 U thrombin (Yuanye, Shanghai, China) to remove the GST tag, and applied to a GSTrap HP column again. The eluted Pn-AqpC-10His was analyzed on a 12% sodium dodecyl sulfate-polyacrylamide gel. LC-MS/MS was implemented to identify Pn-AqpC-10His protein using the Easy-nLC integrated nano-HPLC system (Proxeon) and Q-Extractive mass spectrometer (Thermo). Then fractions with desired protein were stored in aliquots in 10% glycerol at –80°C until use.

For reconstitution of Pn-AqpC into proteoliposomes, the 1:1 mixed lipid 18:1 Cardiolipin (Avanti) and 16:0-18:1 PG (Avanti), the main lipid components of the *S. pneumoniae* cellular membrane (1, 2), and *E. coli* total lipid extract (Avanti) were dissolved in chloroform, dried and resuspended in 100 mM Mops-Na buffer (pH 7.5) containing 2 mM β-mercaptoethanol at concentration of 25 mg/mL. Then the lipid suspensions were passed through the 200 nm polymembrane (Sigma) for 21 times using Mini-Extruder (Avanti). A reconstitution mixture was prepared as previously described (3, 4) by sequentially adding: 100 mM Mops-Na (pH 7.5), 1.0% (wt/vol) OG, purified Pn-AqpC (final concentration 250 µg/mL) and 25 mg/mL liposomes. After removing OG using Bio-beads SM-2 (Biorad), proteoliposomes were collected and reconstitution of Pn-AqpC was examined by running on 12% SDS-PAGE. The proteoliposomes and void liposomes were preserved at -20 °C.

**Determination of culture dissolved oxygen contents.** The culture dissolved O_2_ amounts were measured using OXY Meter (Unisense, Denmark) and Phosphorescent Oxygen Probe (Cayman). OXY Meter measurement of tested strains grown in 40 mL of BHI broth contained in a 50 mL capped centrifuge tube was performed according to the protocols recommended by the supplier. For Phosphorescent Oxygen Probe determination, the mid-exponential cells were collected and re-suspended in the fully aired culture medium containing 10 nM Phosphorescent Oxygen Probe, whose fluorescent signal is quenched by oxygen, and then the mixtures were dispersed into a 96-well plate and sealed with mineral oil. Then the fluorescence intensities were measured as described above. The experiments were executed in triplicate, and each was repeated at least three times independently.

**Determination of excreted and cellular hydrogen peroxide.** The culture H_2_O_2_ was quantified as described previously (5). For determination of H_2_O_2_ produced by pneumococci growing on agar plate, 1.3 mL PBS and 1.2 mL phenol solution containing 2.5 mM 4-amino-antipyrine (4-amino-2,3-dimethyl-1-phenyl-3-pyrazolin-5-one; Sigma) and 0.17 M phenol was added into the plastic petri dish. After 4 min incubation, horseradish peroxidase (Sigma) was added to a final concentration of 500 mU/mL and reaction was proceeded for another 4 min. Cellular H_2_O_2_ was detected using HyPer fluorescence as described previously (6).

**Assay of hydrogen peroxide and NO sensitivity.** For H_2_O_2_ sensitivity assay, the tested strains were statically grown to OD_600_~0.4–0.5, two aliquots were treated with 10 mM H_2_O_2_ for 10 min or not, another was prepulsed with 40 µM of H_2_O_2_ for 20 min prior to 10 mM H_2_O_2_ treatment. NO sensitivity was assayed as described previously (7). Briefly, two aliquots of statically grown cells were resuspended in acidified PBS (pH 4.5) buffer containing 5 mM NaNO_2_ or not for 20 min, another aliquot was prepulsed with 40 µM H_2_O_2_ for 20 min prior to NaNO_2_ treatment. Then bacteria were washed and 10-fold serially diluted, and colony forming units (CFUs) were counted. The survival rates were calculated by dividing the CFUs of H_2_O_2_- or NO-challenged sample over those of the corresponding controls. Experiments were executed in triplicate, and each was repeated at least three times independently.

**Assay of pneumococcus survivability in macrophages.** The mouse leukemia cells of monocyte macrophage Raw 264.7 were purchased from the Cellbank of Chinese Academy of Sciences and cultured in DMEM (HyClone) supplemented with 5% fetal bovine serum (FBS; Invitrogen), penicillin (100 µg/mL) and streptomycin (100 µg/mL) at 37°C with 5% CO_2_. Raw 264.7 cells (1 × 10^5^) were challenged with 1× 10^7^ CFU/mL pneumococcus (multiplicity of infection, 100:1) for 1 h in medium without antibiotic. After removing the bacteria, macrophages were washed and lysed, the number of pneumococci adherent to macrophages were count after 10-fold serial dilution. To quantify the viable pneumococcal cells inside macrophages, the macrophages were incubated in DMEM medium containing antibiotic for additional 1 and 1.5 h, then macrophages were washed twice and lysed, and pneumococcal CFUs were enumerated. Experiments were executed in triplicate, and each was repeated at least three times.

**Determination of the pneumococcal damage to macrophages.** Raw 264.7 macrophages were 1 h infected by various pneumococcus strains (MOI 100:1). After removal of bacteria, macrophages were cultured for another 16 h, and stained with 2 µM [Calcein-AM](http://www.baidu.com/baidu.php?url=af0000KpxzUee8WytQm1nYGhN0OwDOg4Ago_V2diQzd7pWsz0SgPE5gBdsmEFIP9C3wFN9T3i33tcD1Pm07dtZaI3LvVkl2nNRb2Ab0Kj1NGG74PKDdYb3xpH_8wm3eATMEW49W_vQLHPQfWZAwkAF4hZ7HpYC7ujv4dgDsF9jlzehyIgGo0LjZzn1CSZpHBN4oZBOdwx_M6pLqRV_C1UGqinArL.7D_NR2Ar5Od663rj6tVaymYkc6BHswsgFCnECsunMHLhH7enDgQCI6Jsn_I5W_lIL7mhcLYDePh1-3t85R_nYQ7X1Fu8R0.U1Yk0ZDq1eBsSeE8ztjQJtBSzVitsUof0ZKGm1Ys0ZK1pyI85HuWnHnsP1n4nAPhPWmvmymYryDvuhRduynkrHu-njnv0Zfq8SeQsnS3_5EQJtBSzVitsUof0A-V5HczPfKM5gKzm6KdpHdBmy-bIykV0ZKGujYk0APGujY1rjc0UgfqnH0krNtknjDLg1csPWFxnWD1n7t1PW0k0AVG5H00TMfqPHDd0ANGujYkPjfLg1cknHD30AFG5HDdPNtkPH9xnW0Yg1ckPsKVm1Yknj0kg1D3Pj0krHb4rHNxnHnvP1c1n10dPjNxnWb4njDdPj0srNts0Z7spyfqn0Kkmv-b5H00ThIYmyTqn0K9mWYsg100ugFM5H00TZ0qPj04rHRknjRzP0K8IM0qna3snj0snj0sn0KVIZ0qn0KbuAqs5H00ThCqn0KbugmqTAn0uMfqn0KspjYs0Aq15H00mMTqr0K8IjYs0ZPl5fK9TdqGuAnqTZnVmvY0pywW5R9affKYIgnqnW0knWTYnjmznjnknHn3PjnYPsKzug7Y5HDdP16vnWD3rHRdnjb0Tv-b5yPhuW-9ujmknjKhryw-PjD0mLPV5H77PW9KPH6zrDfvn1RzwWT0mynqnfKsUWYs0Z7VIjYs0Z7VT1Ys0ZGY5Hc0UyPxuMFEUHYsg1Kxn7tsg100uA78IyF-gLK_my4GuZnqn7tsg1Kxn7ts0ZK9I7qhUA7M5H00uAPGujYs0ANYpyfqQHD0mgPsmvnqn0KdTA-8mvnqn0KkUymqn0KhmLNY5H00pgPWUjYs0ZGsUZN15H00mywhUA7M5HD0UAuW5H00uAPWujY0mMfqn0KEIjYs0AqzTZfqnanscznsc10WnansQW0snj0snansczns0Z7xIWYsQWbzg108njKxna3sn7tsQWn1g108nHFxna31PsK-XZfqn0KWThnqP1RLn6&word=%E7%BB%86%E8%83%9E%E6%B4%BB%E6%AD%BB%E5%8F%8C%E6%9F%93%E8%AF%95%E5%89%82%E7%9B%92&ck=953.4.62190.0.0.445.149.0&shh=www.baidu.com&sht=site888_3_pg&us=1.0.1.0.1.302.0&bc=110101) and 4.5 µM PI (Sigma Aldrich). Macrophage cells were examined under a confocal laser scanning microscope (Leica TCS SP8, Leica Microsystems), and excitation of 490 nm and emission of 515 nm were used to observe [Calcein-AM](http://www.baidu.com/baidu.php?url=af0000KpxzUee8WytQm1nYGhN0OwDOg4Ago_V2diQzd7pWsz0SgPE5gBdsmEFIP9C3wFN9T3i33tcD1Pm07dtZaI3LvVkl2nNRb2Ab0Kj1NGG74PKDdYb3xpH_8wm3eATMEW49W_vQLHPQfWZAwkAF4hZ7HpYC7ujv4dgDsF9jlzehyIgGo0LjZzn1CSZpHBN4oZBOdwx_M6pLqRV_C1UGqinArL.7D_NR2Ar5Od663rj6tVaymYkc6BHswsgFCnECsunMHLhH7enDgQCI6Jsn_I5W_lIL7mhcLYDePh1-3t85R_nYQ7X1Fu8R0.U1Yk0ZDq1eBsSeE8ztjQJtBSzVitsUof0ZKGm1Ys0ZK1pyI85HuWnHnsP1n4nAPhPWmvmymYryDvuhRduynkrHu-njnv0Zfq8SeQsnS3_5EQJtBSzVitsUof0A-V5HczPfKM5gKzm6KdpHdBmy-bIykV0ZKGujYk0APGujY1rjc0UgfqnH0krNtknjDLg1csPWFxnWD1n7t1PW0k0AVG5H00TMfqPHDd0ANGujYkPjfLg1cknHD30AFG5HDdPNtkPH9xnW0Yg1ckPsKVm1Yknj0kg1D3Pj0krHb4rHNxnHnvP1c1n10dPjNxnWb4njDdPj0srNts0Z7spyfqn0Kkmv-b5H00ThIYmyTqn0K9mWYsg100ugFM5H00TZ0qPj04rHRknjRzP0K8IM0qna3snj0snj0sn0KVIZ0qn0KbuAqs5H00ThCqn0KbugmqTAn0uMfqn0KspjYs0Aq15H00mMTqr0K8IjYs0ZPl5fK9TdqGuAnqTZnVmvY0pywW5R9affKYIgnqnW0knWTYnjmznjnknHn3PjnYPsKzug7Y5HDdP16vnWD3rHRdnjb0Tv-b5yPhuW-9ujmknjKhryw-PjD0mLPV5H77PW9KPH6zrDfvn1RzwWT0mynqnfKsUWYs0Z7VIjYs0Z7VT1Ys0ZGY5Hc0UyPxuMFEUHYsg1Kxn7tsg100uA78IyF-gLK_my4GuZnqn7tsg1Kxn7ts0ZK9I7qhUA7M5H00uAPGujYs0ANYpyfqQHD0mgPsmvnqn0KdTA-8mvnqn0KkUymqn0KhmLNY5H00pgPWUjYs0ZGsUZN15H00mywhUA7M5HD0UAuW5H00uAPWujY0mMfqn0KEIjYs0AqzTZfqnanscznsc10WnansQW0snj0snansczns0Z7xIWYsQWbzg108njKxna3sn7tsQWn1g108nHFxna31PsK-XZfqn0KWThnqP1RLn6&word=%E7%BB%86%E8%83%9E%E6%B4%BB%E6%AD%BB%E5%8F%8C%E6%9F%93%E8%AF%95%E5%89%82%E7%9B%92&ck=953.4.62190.0.0.445.149.0&shh=www.baidu.com&sht=site888_3_pg&us=1.0.1.0.1.302.0&bc=110101) stained live cells and excitation of 535 nm and emission of 617 nm for PI stained dead cells. Two hundred cells within three overlay images of each staining were counted and the macrophage death percentages were calculated by dividing dead cells over the total. Lactate dehydrogenase (LDH) activities were determined using Cytotoxicity Detection kit (Beyotime, Shanghai, China) per the supplier’s recommendation. The results are expressed as the percentages of total cytoplasmic LDH activities of uninfected macrophages lysed in 0.1% triton X-100. Experiments were conducted in triplicate, and each repeated at least three times independently.

**Western blot.** Pneumococcal cells were lysed in RIPA buffer () containing 1 mM PMSF by sonication. The cell wall component, cellular membrane and cytoplasm proteins were separated by centrifugation. Same amounts of proteins were separated by SDS-PAGE and hybridized with HRP-conjugated Anti-GFP-tag mouse monoclonal antibody (Proteintech) or HRP-conjugated anti-His-tag monoclonal antibody (Abmart, Shanghai, China). Western blot signals were detected using the Chemiluminescent Nucleic Acid Detection Module kit (Thermo). The intensities of the target bands were quantified using Image J.

**Heterologous expression of Pn-*aqpC* in *S. cerevisiae*, MbO_2_ determination and GFP observation.** The *S. pneumoniae* R6 Pn-*aqpC* gene containing a 3×Flag tag at 5’ terminus was integrated into plasmid pAG426GAL-ccdB (Addgene). Meanwhile, the whale (*Physeter macrocephalus*) myoglobin (Mb) gene fusing 6×His tag at 3’ terminus was amplified from plasmid pMB413a (Addgene) and integrated into pAG425GAL-ccdB (Addgene). pAG426GAL-ccdB-Pn-*aqpC* and pAG425GAL-ccdB-myo were co-transformed into *S. cerevisiae* INVSc1 using a Yeast transformation kit (Labest Company, Beijing, China) to construct INVSc1-Pn*-aqpC-*myo strain, while pAG426GAL-ccdB and pAG425GAL-ccdB-myo were co-transformed into *S. cerevisiae* INVSc1 to construct INVSc1-myo. Correct transformants were verified by plasmid extraction, PCR, and sequencing.

The MbO_2_ contents in *S. cerevisiae* INVSc1 were determined according to Zwiazek et al. (8) with slight modification. The overnight cultures of INVSc1-Pn*-aqpC-*myo and INVSc1-myo strains in SD-Ura-Leu glucose medium were diluted into fresh SD-Ura-Leu galactose medium to induce the expression of the Pn-*aqpC*-*gfp* and myoglobin genes for 16 h. Then 4 mL of tested yeast cultures at OD_600_ of 1 were harvested, washed and treated with yeast lytic enzyme zymolyase-20T (Zymo, Irvine, CA) at 37 °C, 80 rpm for 3 hr. Then yeast protoplasts were re-suspended in 4 mL of buffer solution (1.2 M sorbitol, 50 mM magnesium acetate, 10 mM CaCl_2_), first de-oxygenated by 7 cycles of vacuuming and N_2_ gas flushing. Next, the de-oxygenated cultures were aerated by bubbling with oxygen pump, and 200 µL cells were dispersed into each well of a 96-well plate. Absorptions of the cell suspension were then scanned from 300 to 650 nm for 7 min at a 60 sec-interval using Synergy H4 Hybrid Multi-Mode Microplate reader (Biotek, Winooski, VT). *S. cerevisiae* INVSc1 was included as a background control. The commercial hemoglobin (Macklin, Shanghai, China) was used as a positive control. The experiments were carried out in triplicate and repeated for three times.

The PCR product of *S. pneumoniae* Pn-*aqpC* gene fusing with green fluorescence protein (sfGFP) gene at 3’ terminus was inserted into the compatible sites on pYES2 vector (Thermofisher, MA, USA), and then transformed into *S. cerevisiae* INVSc1. The correct strains carrying pYES2-Pn-*aqpC*-*gfp* or pYES2 were grown in SD-Ura galactose medium to induce express of the Pn-*aqpC*-*gfp* gene. After 30 min air exposure in the dark, the cells were visualized under a confocal laser scanning microscope (Leica TCS SP8). Excitation was provided at 488 nm, and emission was collected from a range of 500–600 nm.

**Liquid chromatography-tandem mass spectrometry (LC-MS/MS) identification of Pn-AqpC-10His protein.** The target protein gel bands on 12% SDS-PAGE were cut into pieces and washed with MS-grade water for three times. Proteins in the gel were reduced with 25 mM DTT for 45 min at 55 °C; Then DTT was removed, proteins were alkylated with 55 mM iodoacetamide for 30 min in the dark. Upon in-gel digestion with MS-grade trypsin and chymotrypsin (Promega, Fitchburg, WI), LC-MS/MS analysis was implemented with the Easy-nLC integrated nano-HPLC system (Proxeon, Odense, Denmark) and Q-Extractive mass spectrometer (Thermo, Waltham, MA).

**Quantitative RT-PCR.** Total RNA was extracted from the mid-exponential cultures (OD_600_ ~ 0.4 to 0.5) of the tested strains using TRIzol reagent (Invitrogen, Carlsbad, CA) as recommended by the suppliers. After quality confirmation on 1% agarose gel, the RNA extracts were treated with RNase-free DNase (Promega, Madison, WI). cDNAs were generated from 2 µg of total RNA with random primers using Moloney murine leukemia virus reverse transcriptase (Promega, Madison, WI) according to the supplier’s instructions and used for quantitative-PCR (qPCR) amplification with the corresponding primers (Table S2). Amplifications were performed with a Mastercycler ep realplex^2^ (Eppendorf, Germany). To estimate mRNA copies of the target gene, a standard curve for each gene was generated by quantitative PCR using 10-fold serially diluted PCR product as the template. The 16S rRNA gene was used as the biomass reference. The transcript copies of target genes per 1000 16S rRNA copies are shown. All the measurements were done for triplicate samples and repeated at least three times.

**Supplemental references:**

1. Trombe MC, Laneelle MA, Laneelle G. 1979. Lipid composition of aminopterin-resistant and sensitive strains of *Streptococcus pneumoniae*. Effect of aminopterin inhibition. Biochim Biophys Acta 574:290-300.

2. Hosek T, Bougault CM, Lavergne JP, Martinez D, Ayala I, Fenel D, Restelli M, Morlot C, Habenstein B, Grangeasse C, Simorre JP. 2020. Structural features of the interaction of MapZ with FtsZ and membranes in *Streptococcus pneumoniae*. Sci Rep 10:4051.

3. Borgnia MJ, Kozono D, Calamita G, Maloney PC, Agre P. 1999. Functional reconstitution and characterization of AqpZ, the *E. coli* water channel protein. J Mol Biol 291:1169-1179.

4. Borgnia MJ, Agre P. 2001. Reconstitution and functional comparison of purified GlpF and AqpZ, the glycerol and water channels from *Escherichia coli*. Proc Natl Acad Sci U S A 98:2888-2893.

5. Liu L, Tong H, Dong X. 2012. Function of the pyruvate oxidase-lactate oxidase cascade in interspecies competition between *Streptococcus oligofermentans* and *Streptococcus mutans*. Appl Environ Microbiol 78:2120-2127.

6. Tong H, Wang X, Dong Y, Hu Q, Zhao Z, Zhu Y, Dong L, Bai F, Dong X. 2019. A *Streptococcus* aquaporin acts as peroxiporin for efflux of cellular hydrogen peroxide and alleviation of oxidative stress. J Biol Chem 294:4583-4595.

7. Park CY, Kim EH, Choi SY, Tran TD, Kim IH, Kim SN, Pyo S, Rhee DK. 2010. Virulence attenuation of *Streptococcus pneumoniae* *clpP* mutant by sensitivity to oxidative stress in macrophages via an NO-mediated pathway. J Microbiol 48:229-235.

8. Zwiazek JJ, Xu H, Tan X, Navarro-Rodenas A, Morte A. 2017. Significance of oxygen transport through aquaporins. Sci Rep 7:40411.
